# Supplementary material for: From Discrete Complexes to Metal–Organic Layered Materials: Remarkable Hydrogen Bonding Frameworks
Source: Molecules. 2020 Mar 16;25(6):1353. doi: 10.3390/molecules25061353 (PMC7146137; doi:10.3390/molecules25061353)

# checkCIF/PLATON report

Structure factors have been supplied for datablock(s) I

THIS REPORT IS FOR GUIDANCE ONLY. IF USED AS PART OF A REVIEW PROCEDURE FOR PUBLICATION, IT SHOULD NOT REPLACE THE EXPERTISE OF AN EXPERIENCED CRYSTALLOGRAPHIC REFEREE.

No syntax errors found. CIF dictionary Interpreting this report

# Datablock: I

---

```
Bond precision:  C-C = 0.0080 A          Wavelength=0.71073

Cell:           a=17.6784(10)     b=29.6003(18)      c=11.7133(7)
                alpha=90          beta=98.049(4)     gamma=90
Temperature:    150 K

                Calculated                 Reported
Volume          6069.0(6)                  6069.0(6)
Space group     P 21/c                     P 21/c
Hall group      -P 2ybc                    -P 2ybc1
                C8 H26 Mg2 N O14, C8 H5 N  C8 H26 Mg2 N O14, C8 H5 N
Moiety formula  O4, 3(C8 H6 N O4), H12 Mg  O4 3(C8 H6 N O4), H12 Mg
                O6, 10(H2 O                O6, 10(H2 O)
Sum formula     C40 H81 Mg3 N5 O46         C40 H81 Mg3 N5 O46
Mr              1441.03                    1441.03
Dx,g cm-3       1.577                      1.577
Z               4                          4
Mu (mm-1)       0.171                      0.171
F000            3040.0                     3040.0
F000'           3042.66
h,k,lmax        21,36,14                   21,36,14
Nref            11532                      11485
Tmin,Tmax       0.976,0.986                0.975,0.986
Tmin'           0.975

Correction method= MULTI-SCAN

Data completeness= 0.996          Theta(max)= 25.680

R(reflections)= 0.0793( 6035)     wR2(reflections)= 0.2399( 11485)

S = 1.022                 Npar= Npar =1045
```

---

The following ALERTS were generated. Each ALERT has the format
**test-name_ALERT_alert-type_alert-level.**
Click on the hyperlinks for more details of the test.

---

## Alert level A

```
PLAT417_ALERT_2_A Short Inter D-H..H-D       H5W    ..  H47W    ..       1.58 Ang.
PLAT417_ALERT_2_A Short Inter D-H..H-D       H11W   ..  H47W    ..       1.48 Ang.
PLAT417_ALERT_2_A Short Inter D-H..H-D       H12W   ..  H42W    ..       1.55 Ang.
PLAT417_ALERT_2_A Short Inter D-H..H-D       H26W   ..  H49W    ..       1.41 Ang.
```

---

## Alert level B

```
DIFMX01_ALERT_2_B  The maximum difference density is > 0.1*ZMAX*1.00
            _refine_diff_density_max given =      1.307
            Test value =      1.200
PLAT097_ALERT_2_B Large Reported Max.  (Positive) Residual Density       1.31 eA-3
PLAT213_ALERT_2_B Atom N3             has ADP max/min Ratio .....         5.0 oblate
PLAT417_ALERT_2_B Short Inter D-H..H-D       H16W   ..  H50W    ..       2.03 Ang.
PLAT420_ALERT_2_B D-H Without Acceptor       O15W   -   H29W   ...     Please Check
PLAT910_ALERT_3_B Missing # of FCF Reflections Below Th(Min) .....         36 Why ?
```

---

## Alert level C

```
DIFMX02_ALERT_1_C  The maximum difference density is > 0.1*ZMAX*0.75
            The relevant atom site should be identified.
RINTA01_ALERT_3_C  The value of Rint is greater than 0.12
            Rint given   0.147
PLAT020_ALERT_3_C The value of Rint is greater than 0.12 .........      0.147
PLAT094_ALERT_2_C Ratio of Maximum / Minimum Residual Density ....       2.65 Why ?
PLAT213_ALERT_2_C Atom C20            has ADP max/min Ratio .....         3.3 oblate
PLAT213_ALERT_2_C Atom O15            has ADP max/min Ratio .....         3.5 prolat
PLAT213_ALERT_2_C Atom C25            has ADP max/min Ratio .....         3.6 prolat
PLAT250_ALERT_2_C Large U3/U1 Ratio for Average U(i,j) Tensor ....        2.3 Note
PLAT250_ALERT_2_C Large U3/U1 Ratio for Average U(i,j) Tensor ....        2.6 Note
PLAT250_ALERT_2_C Large U3/U1 Ratio for Average U(i,j) Tensor ....        2.2 Note
PLAT314_ALERT_2_C Check Small Angle for H2O: Metal-O10W   -H19W         55.33 Degree
PLAT314_ALERT_2_C Check Small Angle for H2O: Metal-O16W   -H32W         73.10 Degree
PLAT340_ALERT_3_C Low Bond Precision on  C-C Bonds ...............     0.0080 Ang.
PLAT417_ALERT_2_C Short Inter D-H..H-D       H1W    ..  H39W    ..       2.13 Ang.
PLAT417_ALERT_2_C Short Inter D-H..H-D       H16W   ..  H52W    ..       2.13 Ang.
PLAT906_ALERT_3_C Large K value in the Analysis of Variance ......      8.264 Check
PLAT911_ALERT_3_C Missing # FCF Refl Between THmin & STh/L=  0.600         13 Why ?
PLAT975_ALERT_2_C Check Calcd Residual Density  1.10A From    O26W       0.61 eA-3
PLAT975_ALERT_2_C Check Calcd Residual Density  0.85A From      N2       0.55 eA-3
PLAT975_ALERT_2_C Check Calcd Residual Density  1.07A From     O8W       0.54 eA-3
PLAT976_ALERT_2_C Check Calcd Residual Density  0.93A From    O10W      -0.41 eA-3
```

---

## Alert level G

```
FORMU01_ALERT_1_G  There is a discrepancy between the atom counts in the
            _chemical_formula_sum and _chemical_formula_moiety. This is
            usually due to the moiety formula being in the wrong format.
            Atom count from _chemical_formula_sum:   C40 H81 Mg3 N5 O46
            Atom count from _chemical_formula_moiety:C24 H69 Mg3 N3 O77
PLAT002_ALERT_2_G Number of Distance or Angle Restraints on AtSite         97 Note
PLAT003_ALERT_2_G Number of Uiso or Uij Restrained non-H Atoms ...          3 Why ?
PLAT005_ALERT_5_G No _iucr_refine_instructions_details  in the CIF     Please Do !
PLAT042_ALERT_1_G Calc. and Reported MoietyFormula Strings  Differ     Please Check
PLAT066_ALERT_1_G Predicted and Reported Tmin&Tmax Range Identical          ? Check
PLAT072_ALERT_2_G SHELXL First  Parameter in WGHT Unusually Large.       0.11 Why ?
```

```
PLAT083_ALERT_2_G SHELXL Second Parameter in WGHT Unusually Large.      11.07 Why ?
PLAT303_ALERT_2_G Full Occupancy H-Atom  H19W   with # Connections       2.00 Check
PLAT303_ALERT_2_G Full Occupancy H-Atom  H32W   with # Connections       2.00 Check
PLAT790_ALERT_4_G Centre of Gravity not Within Unit Cell: Resd.  #          7 Note
              H2 O
PLAT790_ALERT_4_G Centre of Gravity not Within Unit Cell: Resd.  #          8 Note
              H2 O
PLAT790_ALERT_4_G Centre of Gravity not Within Unit Cell: Resd.  #          9 Note
              H2 O
PLAT860_ALERT_3_G Number of Least-Squares Restraints .............        110 Note
```

---

```
   4 ALERT level A = Most likely a serious problem - resolve or explain
   6 ALERT level B = A potentially serious problem, consider carefully
  21 ALERT level C = Check. Ensure it is not caused by an omission or oversight
  14 ALERT level G = General information/check it is not something unexpected

   4 ALERT type 1 CIF construction/syntax error, inconsistent or missing data
  30 ALERT type 2 Indicator that the structure model may be wrong or deficient
   7 ALERT type 3 Indicator that the structure quality may be low
   3 ALERT type 4 Improvement, methodology, query or suggestion
   1 ALERT type 5 Informative message, check
```

---

# checkCIF publication errors

---

**Alert level A**

```
PUBL004_ALERT_1_A  The contact author's name and address are missing,
            _publ_contact_author_name and _publ_contact_author_address.
PUBL005_ALERT_1_A  _publ_contact_author_email, _publ_contact_author_fax and
            _publ_contact_author_phone are all missing.
            At least one of these should be present.
PUBL006_ALERT_1_A  _publ_requested_journal is missing
            e.g. 'Acta Crystallographica Section C'
PUBL008_ALERT_1_A  _publ_section_title is missing. Title of paper.
PUBL009_ALERT_1_A  _publ_author_name is missing. List of author(s) name(s).
PUBL010_ALERT_1_A  _publ_author_address is missing. Author(s) address(es).
PUBL012_ALERT_1_A  _publ_section_abstract is missing.
            Abstract of paper in English.
```

---

**Alert level G**

```
PUBL017_ALERT_1_G The _publ_section_references section is missing or
            empty.
```

---

```
   7 ALERT level A = Data missing that is essential or data in wrong format
   1 ALERT level G = General alerts. Data that may be required is missing
```

---

**Publication of your CIF**

You should attempt to resolve as many as possible of the alerts in all categories. Often the minor alerts point to easily fixed oversights, errors and omissions in your CIF or refinement strategy, so attention to these fine details can be worthwhile. In order to resolve some of the more serious problems it may be necessary to carry out additional measurements or structure refinements. However, the nature of your study may justify the reported deviations from journal submission requirements and the more serious of these should be commented upon in the discussion or experimental section of a paper or in the "special_details" fields of the CIF. *checkCIF* was carefully designed to identify outliers and unusual parameters, but every test has its limitations and alerts that are not important in a particular case may appear. Conversely, the absence of alerts does not guarantee there are no aspects of the results needing attention. It is up to the individual to critically assess their own results and, if necessary, seek expert advice.

If level A alerts remain, which you believe to be justified deviations, and you intend to submit this CIF for publication in a journal, you should additionally insert an explanation in your CIF using the Validation Reply Form (VRF) below. This will allow your explanation to be considered as part of the review process.

```
# start Validation Reply Form
_vrf_PUBL004_GLOBAL
;
PROBLEM: The contact author’s name and address are missing,
RESPONSE: ...
;
_vrf_PUBL005_GLOBAL
;
PROBLEM: _publ_contact_author_email, _publ_contact_author_fax and
RESPONSE: ...
;
_vrf_PUBL006_GLOBAL
;
PROBLEM: _publ_requested_journal is missing
RESPONSE: ...
;
_vrf_PUBL008_GLOBAL
;
PROBLEM: _publ_section_title is missing. Title of paper.
RESPONSE: ...
;
_vrf_PUBL009_GLOBAL
;
PROBLEM: _publ_author_name is missing. List of author(s) name(s).
RESPONSE: ...
;
_vrf_PUBL010_GLOBAL
;
PROBLEM: _publ_author_address is missing. Author(s) address(es).
RESPONSE: ...
;
_vrf_PUBL012_GLOBAL
;
PROBLEM: _publ_section_abstract is missing.
RESPONSE: ...
;
_vrf_PLAT417_I
;
```

```
PROBLEM: Short Inter D-H..H-D       H5W    ..  H47W    ..       1.58 Ang.
RESPONSE: ...
;
# end Validation Reply Form
```

If you wish to submit your CIF for publication in Acta Crystallographica Section C or E, you should upload your CIF via the web. If your CIF is to form part of a submission to another IUCr journal, you will be asked, either during electronic submission or by the Co-editor handling your paper, to upload your CIF via our web site.

---

## PLATON version of 05/02/2014; check.def file version of 05/02/2014

**Datablock I** - ellipsoid plot

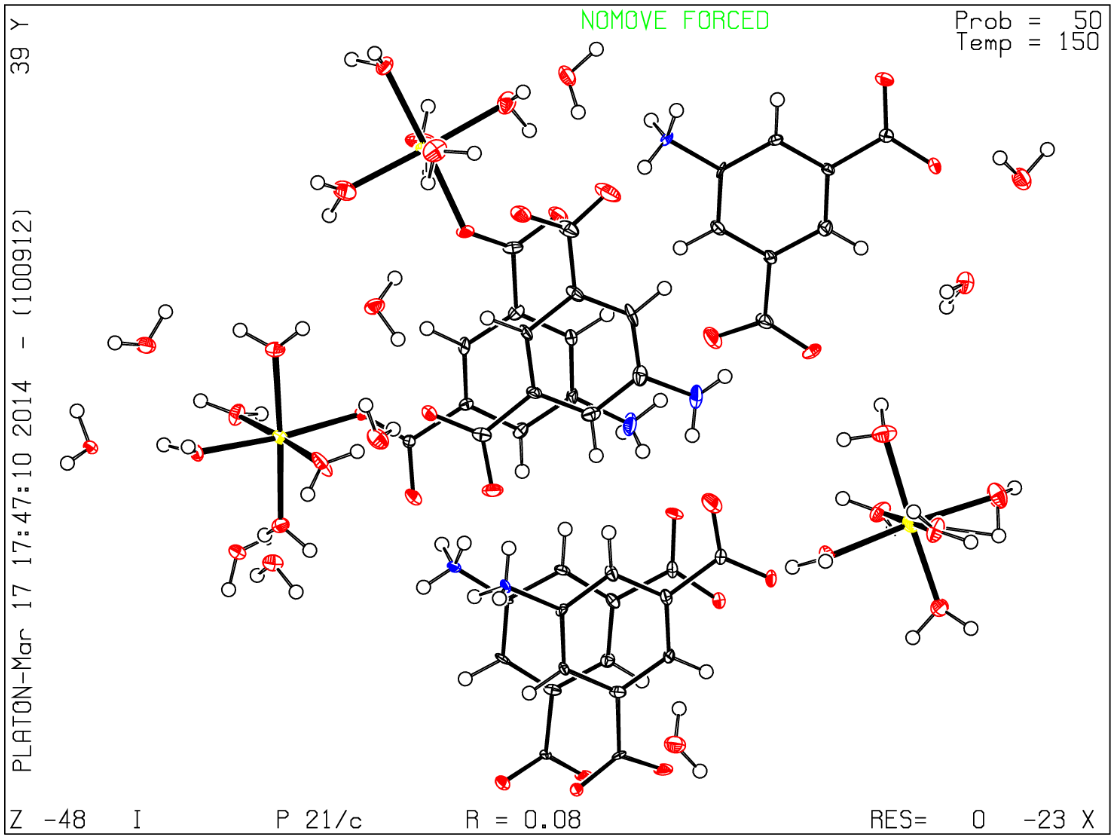

Supplement: Supplementary file 1 [file molecules-25-01353-s001.zip › 1_checkCIF.pdf]
