# Supplementary material for: From Discrete Complexes to Metal–Organic Layered Materials: Remarkable Hydrogen Bonding Frameworks
Source: Molecules. 2020 Mar 16;25(6):1353. doi: 10.3390/molecules25061353 (PMC7146137; doi:10.3390/molecules25061353)
Supplement: Supplementary file 1 [file molecules-25-01353-s001.zip › 2_checkCIF.pdf]

checkCIF () running

Checking for embedded fcf data in CIF ...
Found embedded fcf data in CIF. Extracting fcf data from uploaded CIF, please wait . . . .

checkCIF/PLATON (full publication check)

Structure factors have been supplied for datablock(s) I

THIS REPORT IS FOR GUIDANCE ONLY. IF USED AS PART OF A REVIEW PROCEDURE FOR PUBLICATION, IT SHOULD NOT REPLACE THE EXPERTISE OF AN EXPERIENCED CRYSTALLOGRAPHIC REFEREE.

No syntax errors found.

CIF dictionary
Interpreting this report

Datablock: I

Bond precision:	C-C = 0.0123 A		Wavelength=0.71073
Cell:	a=11.7432(9)	b=47.433(3)	c=13.9297(9)
	alpha=90	beta=97.143(4)	gamma=90
Temperature:	180 K		

	Calculated	Reported
Volume	7698.8(9)	7698.9(10)
Space group	P 21/c	P 21/c
Hall group	-P 2ybc	-P 2ybc
Moiety formula	C40 H34 Mg2 N6 O12, H2 O	C40 H34 Mg2 N6 O12, H2 O
Sum formula	C40 H36 Mg2 N6 O13	C40 H36 Mg2 N6 O13
Mr	857.37	857.37
Dx,g cm-3	1.479	1.479
Z	8	8
Mu (mm-1)	0.141	0.141
F000	3568.0	3568.0
F000'	3570.42	
h,k,lmax	13,52,15	13,52,15
Nref	11043	10997
Tmin,Tmax	0.967,0.996	0.967,0.996
Tmin'	0.956	

Correction method= # Reported T Limits: Tmin=0.967 Tmax=0.996 AbsCorr = MULTI-SCAN

Data completeness= 0.996 Theta(max)= 23.256

R(reflections)= 0.1085(6552) wR2(reflections)= 0.3209(10997)

S = 1.044 Npar= 1178

The following ALERTS were generated. Each ALERT has the format
test-name_ALERT_alert-type_alert-level.
Click on the hyperlinks for more details of the test.

Alert level B

THETM01_ALERT_3_B	The value of sine(theta_max)/wavelength is less than 0.575		
	Calculated sin(theta_max)/wavelength = 0.5555		
PLAT023_ALERT_3_B	Resolution (too) Low [sin(theta)/Lambda < 0.6]..	23.26	Degree
PLAT213_ALERT_2_B	Atom C7 has ADP max/min Ratio	4.4	prolat
PLAT213_ALERT_2_B	Atom C34 has ADP max/min Ratio	4.5	prolat

```
PLAT220_ALERT_2_B Non-Solvent Resd 1   C   Ueq(max)/Ueq(min) Range        6.2 Ratio
PLAT340_ALERT_3_B Low Bond Precision on  C-C Bonds ...............     0.0123 Ang.
PLAT414_ALERT_2_B Short Intra D-H..H-X        H12W    ..  H52      ..       1.89 Ang.
PLAT417_ALERT_2_B Short Inter D-H..H-D        H1W     ..  H20W     ..       2.08 Ang.
```

And 3 other PLAT417 Alerts

```
PLAT417_ALERT_2_B Short Inter D-H..H-D        H3A     ..  H33A     ..       1.98 Ang.
PLAT417_ALERT_2_B Short Inter D-H..H-D        H16W    ..  H17W     ..       2.04 Ang.
PLAT417_ALERT_2_B Short Inter D-H..H-D        H16W    ..  H18W     ..       2.09 Ang.
PLAT420_ALERT_2_B D-H Without Acceptor        O6W     --  H12W     ...     Please Check
```

Alert level C

```
DIFMN02_ALERT_2_C  The minimum difference density is DIFMN03_ALERT_1_C  The minimum
difference density is REFNR01_ALERT_3_C  Ratio of reflections to parameters is
RFACG01_ALERT_3_C  The value of the R factor is > 0.10
            R factor given    0.108
RFACR01_ALERT_3_C  The value of the weighted R factor is > 0.25
            Weighted R factor given   0.321
PLAT082_ALERT_2_C High R1 Value ..................................       0.11 Report
PLAT084_ALERT_3_C High wR2 Value (i.e. > 0.25) ...................       0.32 Report
PLAT088_ALERT_3_C Poor Data / Parameter Ratio ....................       9.37 Note
PLAT098_ALERT_2_C Large Reported Min.  (Negative) Residual Density      -1.10 eA-3
PLAT213_ALERT_2_C Atom O4W             has ADP max/min Ratio .....        3.2 prolat
```

And 9 other PLAT213 Alerts

```
PLAT213_ALERT_2_C Atom O6              has ADP max/min Ratio .....        3.4 prolat
PLAT213_ALERT_2_C Atom C5              has ADP max/min Ratio .....        3.2 prolat
PLAT213_ALERT_2_C Atom C8              has ADP max/min Ratio .....        3.1 prolat
PLAT213_ALERT_2_C Atom C9              has ADP max/min Ratio .....        3.2 prolat
PLAT213_ALERT_2_C Atom C10             has ADP max/min Ratio .....        3.3 prolat
PLAT213_ALERT_2_C Atom C11             has ADP max/min Ratio .....        3.3 prolat
PLAT213_ALERT_2_C Atom C16             has ADP max/min Ratio .....        3.3 oblate
PLAT213_ALERT_2_C Atom C35             has ADP max/min Ratio .....        3.5 prolat
PLAT213_ALERT_2_C Atom C75             has ADP max/min Ratio .....        3.4 prolat
PLAT220_ALERT_2_C Non-Solvent Resd 2   C   Ueq(max)/Ueq(min) Range        4.0 Ratio
PLAT222_ALERT_3_C Non-Solvent Resd 1   H Uiso(max)/Uiso(min) Range        5.2 Ratio
PLAT234_ALERT_4_C Large Hirshfeld Difference C75     --  C78      ..       0.18 Ang.
PLAT234_ALERT_4_C Large Hirshfeld Difference C76     --  C77      ..       0.17 Ang.
PLAT250_ALERT_2_C Large U3/U1 Ratio for Average U(i,j) Tensor ....        2.3 Note
PLAT420_ALERT_2_C D-H Without Acceptor        N4      --  H3A      ...     Please Check
PLAT420_ALERT_2_C D-H Without Acceptor        N9      --  H6A      ...     Please Check
```

Alert level G

```
PLAT002_ALERT_2_G Number of Distance or Angle Restraints on AtSite        39 Note
PLAT003_ALERT_2_G Number of Uiso or Uij Restrained non-H Atoms ...         1 Report
PLAT007_ALERT_5_G Number of Unrefined Donor-H Atoms .............         2 Report
PLAT066_ALERT_1_G Predicted and Reported Tmin&Tmax Range Identical         ? Check
PLAT072_ALERT_2_G SHELXL First  Parameter in WGHT  Unusually Large      0.17 Report
PLAT083_ALERT_2_G SHELXL Second Parameter in WGHT  Unusually Large     35.63 Why ?
PLAT172_ALERT_4_G The CIF-Embedded .res File Contains DFIX Records        26 Report
PLAT186_ALERT_4_G The CIF-Embedded .res File Contains ISOR Records         1 Report
PLAT333_ALERT_2_G Check Large Av C6-Ring C-C Dist. C4      -C9          1.42 Ang.
PLAT790_ALERT_4_G Centre of Gravity not Within Unit Cell: Resd.  #         2 Note
              C40 H34 Mg2 N6 O12
PLAT790_ALERT_4_G Centre of Gravity not Within Unit Cell: Resd.  #         4 Note
              H2 O
PLAT860_ALERT_3_G Number of Least-Squares Restraints .............        45 Note
```

```
  0 ALERT level A = Most likely a serious problem - resolve or explain
 12 ALERT level B = A potentially serious problem, consider carefully
 26 ALERT level C = Check. Ensure it is not caused by an omission or oversight
 12 ALERT level G = General information/check it is not something unexpected

  2 ALERT type 1 CIF construction/syntax error, inconsistent or missing data
 31 ALERT type 2 Indicator that the structure model may be wrong or deficient
```

```
 10 ALERT type 3 Indicator that the structure quality may be low
  6 ALERT type 4 Improvement, methodology, query or suggestion
  1 ALERT type 5 Informative message, check
```

checkCIF publication errors

Alert level A

```
PUBL004_ALERT_1_A  The contact author's name and address are missing,
            _publ_contact_author_name and _publ_contact_author_address.
PUBL005_ALERT_1_A  _publ_contact_author_email, _publ_contact_author_fax and
            _publ_contact_author_phone are all missing.
            At least one of these should be present.
PUBL006_ALERT_1_A  _publ_requested_journal is missing
            e.g. 'Acta Crystallographica Section C'
PUBL008_ALERT_1_A  _publ_section_title is missing. Title of paper.
PUBL009_ALERT_1_A  _publ_author_name is missing. List of author(s) name(s).
PUBL010_ALERT_1_A  _publ_author_address is missing. Author(s) address(es).
PUBL012_ALERT_1_A  _publ_section_abstract is missing.
            Abstract of paper in English.
```

Alert level G

```
PUBL017_ALERT_1_G The _publ_section_references section is missing or
            empty.
```

```
  7 ALERT level A = Data missing that is essential or data in wrong format
  1 ALERT level G = General alerts. Data that may be required is missing
```

Publication of your CIF

You should attempt to resolve as many as possible of the alerts in all categories. Often the minor alerts point to easily fixed oversights, errors and omissions in your CIF or refinement strategy, so attention to these fine details can be worthwhile. In order to resolve some of the more serious problems it may be necessary to carry out additional measurements or structure refinements. However, the nature of your study may justify the reported deviations from journal submission requirements and the more serious of these should be commented upon in the discussion or experimental section of a paper or in the "special_details" fields of the CIF. *checkCIF* was carefully designed to identify outliers and unusual parameters, but every test has its limitations and alerts that are not important in a particular case may appear. Conversely, the absence of alerts does not guarantee there are no aspects of the results needing attention. It is up to the individual to critically assess their own results and, if necessary, seek expert advice.
If level A alerts remain, which you believe to be justified deviations, and you intend to submit this CIF for publication in a journal, you should additionally insert an explanation in your CIF using the Validation Reply Form (VRF) below. This will allow your explanation to be considered as part of the review process.

Validation response form

Please find below a validation response form (VRF) that can be filled in and pasted into your CIF.

```
# start Validation Reply Form
_vrf_PUBL004_GLOBAL
;
PROBLEM: The contact author's name and address are missing,
RESPONSE: ...
;
_vrf_PUBL005_GLOBAL
;
PROBLEM: _publ_contact_author_email, _publ_contact_author_fax and
RESPONSE: ...
;
_vrf_PUBL006_GLOBAL
;
PROBLEM: _publ_requested_journal is missing
RESPONSE: ...
;
_vrf_PUBL008_GLOBAL
;
PROBLEM: _publ_section_title is missing. Title of paper.
RESPONSE: ...
;
_vrf_PUBL009_GLOBAL
;
PROBLEM: _publ_author_name is missing. List of author(s) name(s).
```

```
RESPONSE: ...
;
_vrf_PUBL010_GLOBAL
;
PROBLEM: _publ_author_address is missing. Author(s) address(es).
RESPONSE: ...
;
_vrf_PUBL012_GLOBAL
;
PROBLEM: _publ_section_abstract is missing.
RESPONSE: ...
;
# end Validation Reply Form
```

If you wish to submit your CIF for publication in Acta Crystallographica Section C or E, you should upload your CIF via the web. If you wish to submit your CIF for publication in IUCrData you should upload your CIF via the web. If your CIF is to form part of a submission to another IUCr journal, you will be asked, either during electronic submission or by the Co-editor handling your paper, to upload your CIF via our web site.

PLATON version of 11/08/2016; check.def file version of 04/08/2016

Datablock I - ellipsoid plot

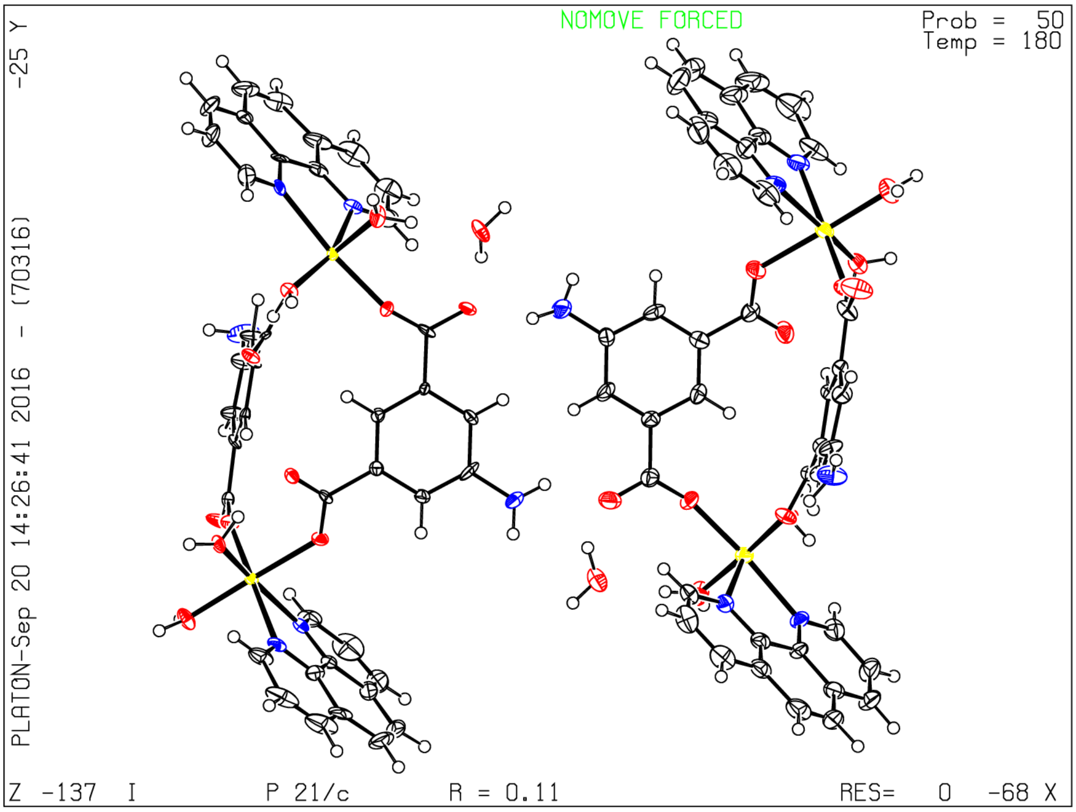

Download CIF editor (publCIF) from the IUCr
Download CIF editor (enCIFer) from the CCDC
Test a new CIF entry
