# Supplementary material for: From Discrete Complexes to Metal–Organic Layered Materials: Remarkable Hydrogen Bonding Frameworks
Source: Molecules. 2020 Mar 16;25(6):1353. doi: 10.3390/molecules25061353 (PMC7146137; doi:10.3390/molecules25061353)

checkCIF/PLATON report

Structure factors have been supplied for datablock(s) I

THIS REPORT IS FOR GUIDANCE ONLY. IF USED AS PART OF A REVIEW PROCEDURE FOR PUBLICATION, IT SHOULD NOT REPLACE THE EXPERTISE OF AN EXPERIENCED CRYSTALLOGRAPHIC REFEREE.

No syntax errors found. CIF dictionary Interpreting this report

Datablock: I

```
Bond precision:  C-C = 0.0021 A          Wavelength=0.71073

Cell:           a=8.1375(9)       b=10.2726(10)    c=13.3429(14)
                alpha=74.135(5)   beta=79.343(5)   gamma=84.520(5)
Temperature:    150 K

                Calculated                 Reported
Volume          1053.19(19)                1053.19(19)
Space group     P -1                       P -1
Hall group      -P 1                       -P 1
Moiety formula  C8 H9 Ca N O6, H2 O        C8 H9 Ca N O6, H2 O
Sum formula     C8 H11 Ca N O7             C8 H11 Ca N O7
Mr              273.26                     273.26
Dx,g cm-3       1.723                      1.723
Z               4                          4
Mu (mm-1)       0.621                      0.621
F000            568.0                      568.0
F000'           569.27
h,k,lmax        9,12,15                    9,12,15
Nref            3714                       3680
Tmin,Tmax       0.928,0.969                0.902,0.970
Tmin'           0.900

Correction method= MULTI-SCAN

Data completeness= 0.991          Theta(max)= 25.030

R(reflections)= 0.0292( 2688)     wR2(reflections)= 0.0899( 3680)

S = 1.093                  Npar= Npar = 355
```

The following ALERTS were generated. Each ALERT has the format
test-name_ALERT_alert-type_alert-level.
Click on the hyperlinks for more details of the test.

Alert level B

```
PLAT417_ALERT_2_B Short Inter D-H..H-D       H1W    ..  H9W     ..       1.80 Ang.
PLAT910_ALERT_3_B Missing # of FCF Reflections Below Th(Min) .....         12 Why ?
```

Alert level C

```
PLAT414_ALERT_2_C Short Intra D-H..H-X       H3W    ..  H7      ..       1.97 Ang.
PLAT417_ALERT_2_C Short Inter D-H..H-D       H5W    ..  H11W    ..       2.13 Ang.
PLAT906_ALERT_3_C Large K value in the Analysis of Variance ......      2.845 Check
PLAT911_ALERT_3_C Missing # FCF Refl Between THmin & STh/L=  0.595         21 Why ?
```

Alert level G

```
PLAT002_ALERT_2_G Number of Distance or Angle Restraints on AtSite         24 Note
PLAT004_ALERT_5_G Polymeric Structure Found with Dimension .......          1 Info
PLAT004_ALERT_5_G Polymeric Structure Found with Dimension .......          1 Info
PLAT005_ALERT_5_G No _iucr_refine_instructions_details  in the CIF     Please Do !
PLAT066_ALERT_1_G Predicted and Reported Tmin&Tmax Range Identical          ? Check
PLAT110_ALERT_2_G ADDSYM Detects Potential Lattice Centering .....          ? Check
PLAT112_ALERT_2_G ADDSYM Detects Additional (Pseudo) Symm. Elem...          Z Check
PLAT113_ALERT_2_G ADDSYM Suggests Possible Pseudo/New Space-group.        P-1 Check
PLAT154_ALERT_1_G The su's on the Cell Angles are Equal ..........    0.00500 Degree
PLAT764_ALERT_4_G Overcomplete CIF Bond List Detected (Rep/Expd) .       1.26 Ratio
PLAT790_ALERT_4_G Centre of Gravity not Within Unit Cell: Resd.  #          2 Note
              C8 H9 Ca N O6
PLAT790_ALERT_4_G Centre of Gravity not Within Unit Cell: Resd.  #          3 Note
              H2 O
PLAT790_ALERT_4_G Centre of Gravity not Within Unit Cell: Resd.  #          4 Note
              H2 O
PLAT860_ALERT_3_G Number of Least-Squares Restraints .............         22 Note
PLAT909_ALERT_3_G Percentage of Observed Data at Theta(Max) still          59 %
```

---

0 **ALERT level A** = Most likely a serious problem - resolve or explain
2 **ALERT level B** = A potentially serious problem, consider carefully
4 **ALERT level C** = Check. Ensure it is not caused by an omission or oversight
15 **ALERT level G** = General information/check it is not something unexpected

2 ALERT type 1 CIF construction/syntax error, inconsistent or missing data
7 ALERT type 2 Indicator that the structure model may be wrong or deficient
5 ALERT type 3 Indicator that the structure quality may be low
4 ALERT type 4 Improvement, methodology, query or suggestion
3 ALERT type 5 Informative message, check

---

checkCIF publication errors

Alert level A

```
PUBL004_ALERT_1_A  The contact author's name and address are missing,
            _publ_contact_author_name and _publ_contact_author_address.
PUBL005_ALERT_1_A  _publ_contact_author_email, _publ_contact_author_fax and
            _publ_contact_author_phone are all missing.
            At least one of these should be present.
PUBL006_ALERT_1_A  _publ_requested_journal is missing
            e.g. 'Acta Crystallographica Section C'
PUBL008_ALERT_1_A  _publ_section_title is missing. Title of paper.
PUBL009_ALERT_1_A  _publ_author_name is missing. List of author(s) name(s).
PUBL010_ALERT_1_A  _publ_author_address is missing. Author(s) address(es).
```

```
PUBL012_ALERT_1_A  _publ_section_abstract is missing.
            Abstract of paper in English.
```

Alert level G

```
PUBL017_ALERT_1_G The _publ_section_references section is missing or
            empty.
```

---

7 **ALERT level A** = Data missing that is essential or data in wrong format
1 **ALERT level G** = General alerts. Data that may be required is missing

---

Publication of your CIF

You should attempt to resolve as many as possible of the alerts in all categories. Often the minor alerts point to easily fixed oversights, errors and omissions in your CIF or refinement strategy, so attention to these fine details can be worthwhile. In order to resolve some of the more serious problems it may be necessary to carry out additional measurements or structure refinements. However, the nature of your study may justify the reported deviations from journal submission requirements and the more serious of these should be commented upon in the discussion or experimental section of a paper or in the "special_details" fields of the CIF. *checkCIF* was carefully designed to identify outliers and unusual parameters, but every test has its limitations and alerts that are not important in a particular case may appear. Conversely, the absence of alerts does not guarantee there are no aspects of the results needing attention. It is up to the individual to critically assess their own results and, if necessary, seek expert advice.

If level A alerts remain, which you believe to be justified deviations, and you intend to submit this CIF for publication in a journal, you should additionally insert an explanation in your CIF using the Validation Reply Form (VRF) below. This will allow your explanation to be considered as part of the review process.

```
# start Validation Reply Form
_vrf_PUBL004_GLOBAL
;
PROBLEM: The contact author’s name and address are missing,
RESPONSE: ...
;
_vrf_PUBL005_GLOBAL
;
PROBLEM: _publ_contact_author_email, _publ_contact_author_fax and
RESPONSE: ...
;
_vrf_PUBL006_GLOBAL
;
PROBLEM: _publ_requested_journal is missing
RESPONSE: ...
;
_vrf_PUBL008_GLOBAL
;
PROBLEM: _publ_section_title is missing. Title of paper.
RESPONSE: ...
;
_vrf_PUBL009_GLOBAL
;
PROBLEM: _publ_author_name is missing. List of author(s) name(s).
RESPONSE: ...
```

```
;
_vrf_PUBL010_GLOBAL
;
PROBLEM: _publ_author_address is missing. Author(s) address(es).
RESPONSE: ...
;
_vrf_PUBL012_GLOBAL
;
PROBLEM: _publ_section_abstract is missing.
RESPONSE: ...
;
# end Validation Reply Form
```

If you wish to submit your CIF for publication in Acta Crystallographica Section C or E, you should upload your CIF via the web. If your CIF is to form part of a submission to another IUCr journal, you will be asked, either during electronic submission or by the Co-editor handling your paper, to upload your CIF via our web site.

PLATON version of 05/02/2014; check.def file version of 05/02/2014

Datablock I - ellipsoid plot

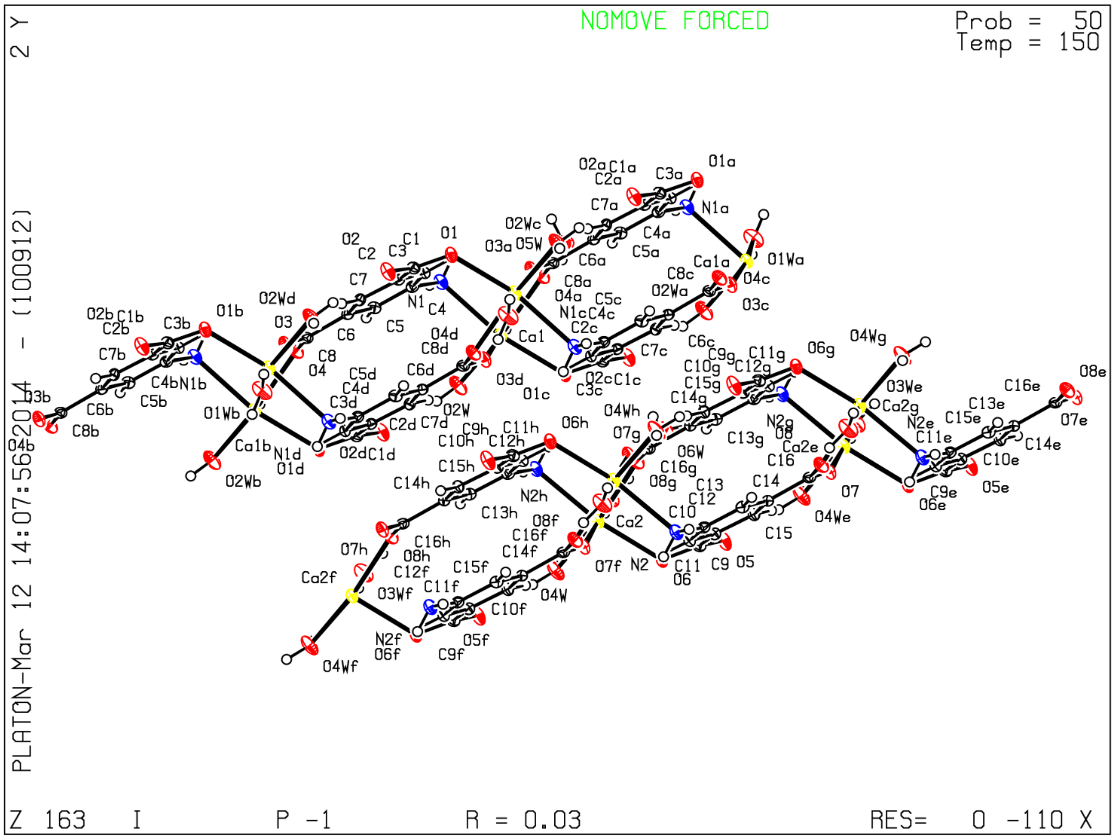

Supplement: Supplementary file 1 [file molecules-25-01353-s001.zip › 3_checkCIF.pdf]
