# Supplementary material for: From Discrete Complexes to Metal–Organic Layered Materials: Remarkable Hydrogen Bonding Frameworks
Source: Molecules. 2020 Mar 16;25(6):1353. doi: 10.3390/molecules25061353 (PMC7146137; doi:10.3390/molecules25061353)

checkCIF/PLATON report

Structure factors have been supplied for datablock(s) I

THIS REPORT IS FOR GUIDANCE ONLY. IF USED AS PART OF A REVIEW PROCEDURE FOR PUBLICATION, IT SHOULD NOT REPLACE THE EXPERTISE OF AN EXPERIENCED CRYSTALLOGRAPHIC REFEREE.

No syntax errors found.   CIF dictionary   Interpreting this report

Datablock: I

```
Bond precision:  C-C = 0.0094 A          Wavelength=0.71073

Cell:          a=10.9458(4)      b=11.9419(4)     c=13.4085(5)
               alpha=64.178(1)   beta=79.870(2)   gamma=77.179(1)
Temperature:   150 K
```

	Calculated	Reported
Volume	1532.06(10)	1532.06(10)
Space group	P 1	P 1
Hall group	P 1	P 1
Moiety formula	C40 H40 Ba2 N6 O15, 2(C12 H8 N2), 2(H2 O)	C40 H40 Ba2 N6 O15, 2(C12 H8 N2), 2(H2 O)
Sum formula	C64 H60 Ba2 N10 O17	C64 H60 Ba2 N10 O17
Mr	1515.88	1515.90
Dx,g cm-3	1.643	1.643
Z	1	1
Mu (mm-1)	1.359	1.359
F000	762.0	762.0
F000’	761.68	
h,k,lmax	14,15,17	14,15,17
Nref	14070[7035]	13072
Tmin,Tmax	0.770,0.873	0.763,0.876
Tmin’	0.752	

```
Correction method= MULTI-SCAN

Data completeness= 1.86/0.93      Theta(max)= 27.480

R(reflections)= 0.0296( 12197)    wR2(reflections)= 0.0597( 13072)

S = 1.041                   Npar= Npar = 904
```

The following ALERTS were generated. Each ALERT has the format
test-name_ALERT_alert-type_alert-level.
Click on the hyperlinks for more details of the test.

Alert level A

```
PLAT417_ALERT_2_A Short Inter D-H..H-D       H14W   ..  H15W    ..       1.40 Ang.
PLAT924_ALERT_1_A The Reported and Calculated Rho(min) Differ by .       8.45 eA-3
PLAT927_ALERT_1_A Reported and Calculated  wR2 Differ by .........    -0.0579 Check
PLAT974_ALERT_2_A Check Calcd Negative Residual Density on     Ba2      -8.86 eA-3
PLAT974_ALERT_2_A Check Calcd Negative Residual Density on     Ba1      -8.69 eA-3
```

Alert level B

```
PLAT420_ALERT_2_B D-H Without Acceptor       O1W    -   H2W    ...     Please Check
PLAT420_ALERT_2_B D-H Without Acceptor       O3W    -   H6W    ...     Please Check
PLAT420_ALERT_2_B D-H Without Acceptor       O4W    -   H7W    ...     Please Check
PLAT420_ALERT_2_B D-H Without Acceptor       O5W    -   H10W   ...     Please Check
PLAT420_ALERT_2_B D-H Without Acceptor       O8W    -   H16W   ...     Please Check
PLAT910_ALERT_3_B Missing # of FCF Reflections Below Th(Min) .....         17 Why ?
PLAT926_ALERT_1_B Reported and Calculated   R1 Differ by .........    -0.0244 Check
PLAT928_ALERT_1_B Reported and Calculated    S value   Differ by .     -1.008
```

Alert level C

```
PLAT036_ALERT_1_C No s.u. Given for Flack Parameter ..............     Please Do !
PLAT090_ALERT_3_C Poor Data / Parameter Ratio (Zmax > 18) ........       7.74 Note
PLAT094_ALERT_2_C Ratio of Maximum / Minimum Residual Density ....       2.06 Why ?
PLAT213_ALERT_2_C Atom N1               has ADP max/min Ratio .....       3.1 prolat
PLAT213_ALERT_2_C Atom C61              has ADP max/min Ratio .....       3.8 prolat
PLAT220_ALERT_2_C Large Non-Solvent  N     Ueq(max)/Ueq(min) Range        3.5 Ratio
PLAT222_ALERT_3_C Large Non-Solvent    H    Uiso(max)/Uiso(min) ..        5.5 Ratio
PLAT241_ALERT_2_C High      Ueq as Compared to Neighbors for .....        C60 Check
PLAT250_ALERT_2_C Large U3/U1 Ratio for Average U(i,j) Tensor ....        3.0 Note
PLAT314_ALERT_2_C Check Small Angle for H2O: Metal-O1W    -H2W          84.22 Degree
PLAT314_ALERT_2_C Check Small Angle for H2O: Metal-O3W    -H6W          88.32 Degree
PLAT314_ALERT_2_C Check Small Angle for H2O: Metal-O7W    -H14W         80.69 Degree
PLAT342_ALERT_3_C Low Bond Precision on  C-C Bonds ...............     0.0094 Ang.
PLAT369_ALERT_2_C Long   C(sp2)-C(sp2) Bond  C9     -   C10    ...       1.55 Ang.
PLAT417_ALERT_2_C Short Inter D-H..H-D       H5W    ..  H17W    ..       2.12 Ang.
PLAT480_ALERT_4_C Long H...A H-Bond Reported H2W    ..  O2W     ..       2.61 Ang.
PLAT480_ALERT_4_C Long H...A H-Bond Reported H3W    ..  O1W     ..       2.66 Ang.
PLAT480_ALERT_4_C Long H...A H-Bond Reported H5W    ..  N9      ..       2.68 Ang.
PLAT480_ALERT_4_C Long H...A H-Bond Reported H9W    ..  N10     ..       2.70 Ang.
PLAT480_ALERT_4_C Long H...A H-Bond Reported H14W   ..  O4W     ..       2.63 Ang.
PLAT480_ALERT_4_C Long H...A H-Bond Reported H16W   ..  O2W     ..       2.65 Ang.
PLAT911_ALERT_3_C Missing # FCF Refl Between THmin & STh/L=  0.600         17 Why ?
PLAT913_ALERT_3_C Missing # of Very Strong Reflections in FCF ....          2 Note
PLAT915_ALERT_3_C Low Friedel Pair Coverage ......................         86 %
PLAT975_ALERT_2_C Check Calcd Residual Density  0.82A From      O2       0.55 eA-3
```

Alert level G

```
PLAT002_ALERT_2_G Number of Distance or Angle Restraints on AtSite         33 Note
PLAT004_ALERT_5_G Polymeric Structure Found with Dimension .......          1 Info
PLAT005_ALERT_5_G No _iucr_refine_instructions_details  in the CIF     Please Do !
PLAT066_ALERT_1_G Predicted and Reported Tmin&Tmax Range Identical          ? Check
PLAT111_ALERT_2_G ADDSYM Detects (Pseudo) Centre of Symmetry .....         81 %Fit
PLAT335_ALERT_2_G Check Large C6 Ring    C-C Range C56    -C61           0.16 Ang.
PLAT710_ALERT_4_G Delete 1-2-3 or 2-3-4 Linear Torsion Angle ... #        210 Do !
            O7  -BA2 -O6  -C9     -8.80  1.00   1.655   1.555   1.555   1.555
PLAT710_ALERT_4_G Delete 1-2-3 or 2-3-4 Linear Torsion Angle ... #        317 Do !
            BA2 -C16 -C12 -C11  -119.10  1.90   1.455   1.555   1.555   1.555
PLAT710_ALERT_4_G Delete 1-2-3 or 2-3-4 Linear Torsion Angle ... #        320 Do !
            BA2 -C16 -C12 -C13    57.00  2.00   1.455   1.555   1.555   1.555
PLAT790_ALERT_4_G Centre of Gravity not Within Unit Cell: Resd.  #          3 Note
```

```
              C12 H8 N2
PLAT790_ALERT_4_G Centre of Gravity not Within Unit Cell: Resd.  #          5 Note
              H2 O
PLAT860_ALERT_3_G Number of Least-Squares Restraints .............         36 Note
PLAT912_ALERT_4_G Missing # of FCF Reflections Above STh/L=  0.600         12 Note
PLAT916_ALERT_2_G Hooft y and Flack x Parameter values differ by .       0.31 Check
```

---

5 **ALERT level A** = Most likely a serious problem - resolve or explain
8 **ALERT level B** = A potentially serious problem, consider carefully
25 **ALERT level C** = Check. Ensure it is not caused by an omission or oversight
14 **ALERT level G** = General information/check it is not something unexpected

6 ALERT type 1 CIF construction/syntax error, inconsistent or missing data
24 ALERT type 2 Indicator that the structure model may be wrong or deficient
8 ALERT type 3 Indicator that the structure quality may be low
12 ALERT type 4 Improvement, methodology, query or suggestion
2 ALERT type 5 Informative message, check

---

checkCIF publication errors

Alert level A

```
PUBL004_ALERT_1_A  The contact author's name and address are missing,
            _publ_contact_author_name and _publ_contact_author_address.
PUBL005_ALERT_1_A  _publ_contact_author_email, _publ_contact_author_fax and
            _publ_contact_author_phone are all missing.
            At least one of these should be present.
PUBL006_ALERT_1_A  _publ_requested_journal is missing
            e.g. 'Acta Crystallographica Section C'
PUBL008_ALERT_1_A  _publ_section_title is missing. Title of paper.
PUBL009_ALERT_1_A  _publ_author_name is missing. List of author(s) name(s).
PUBL010_ALERT_1_A  _publ_author_address is missing. Author(s) address(es).
PUBL012_ALERT_1_A  _publ_section_abstract is missing.
            Abstract of paper in English.
```

Alert level G

```
PUBL017_ALERT_1_G The _publ_section_references section is missing or
            empty.
```

---

7 **ALERT level A** = Data missing that is essential or data in wrong format
1 **ALERT level G** = General alerts. Data that may be required is missing

---

Publication of your CIF

You should attempt to resolve as many as possible of the alerts in all categories. Often the minor alerts point to easily fixed oversights, errors and omissions in your CIF or refinement strategy, so attention to these fine details can be worthwhile. In order to resolve some of the more serious problems it may be necessary to carry out additional measurements or structure refinements. However, the nature of your study may justify the reported deviations from journal submission requirements and the more serious of these should be commented upon in the discussion or experimental section of a paper or in the "special_details" fields of the CIF. *checkCIF* was carefully designed to identify outliers and unusual parameters, but every test has its limitations and alerts that are not important in a particular case may appear. Conversely, the absence of alerts does not guarantee there are no aspects of the results needing attention. It is up to the individual to critically assess their own results and, if necessary, seek expert advice.

If level A alerts remain, which you believe to be justified deviations, and you intend to submit this CIF for publication in a journal, you should additionally insert an explanation in your CIF using the Validation Reply Form (VRF) below. This will allow your explanation to be considered as part of the review process.

```
# start Validation Reply Form
_vrf_PUBL004_GLOBAL
;
PROBLEM: The contact author’s name and address are missing,
RESPONSE: ...
;
_vrf_PUBL005_GLOBAL
;
PROBLEM: _publ_contact_author_email, _publ_contact_author_fax and
RESPONSE: ...
;
_vrf_PUBL006_GLOBAL
;
PROBLEM: _publ_requested_journal is missing
RESPONSE: ...
;
_vrf_PUBL008_GLOBAL
;
PROBLEM: _publ_section_title is missing. Title of paper.
RESPONSE: ...
;
_vrf_PUBL009_GLOBAL
;
PROBLEM: _publ_author_name is missing. List of author(s) name(s).
RESPONSE: ...
;
_vrf_PUBL010_GLOBAL
;
PROBLEM: _publ_author_address is missing. Author(s) address(es).
RESPONSE: ...
;
_vrf_PUBL012_GLOBAL
;
PROBLEM: _publ_section_abstract is missing.
RESPONSE: ...
;
_vrf_PLAT417_I
;
```

```
PROBLEM: Short Inter D-H..H-D       H14W   ..  H15W    ..       1.40 Ang.
RESPONSE: ...
;
_vrf_PLAT924_I
;
PROBLEM: The Reported and Calculated Rho(min) Differ by .       8.45 eA-3
RESPONSE: ...
;
_vrf_PLAT927_I
;
PROBLEM: Reported and Calculated  wR2 Differ by .........    -0.0579 Check
RESPONSE: ...
;
_vrf_PLAT974_I
;
PROBLEM: Check Calcd Negative Residual Density on     Ba2      -8.86 eA-3
RESPONSE: ...
;
# end Validation Reply Form
```

If you wish to submit your CIF for publication in Acta Crystallographica Section C or E, you should upload your CIF via the web. If your CIF is to form part of a submission to another IUCr journal, you will be asked, either during electronic submission or by the Co-editor handling your paper, to upload your CIF via our web site.

PLATON version of 05/02/2014; check.def file version of 05/02/2014

Datablock I - ellipsoid plot

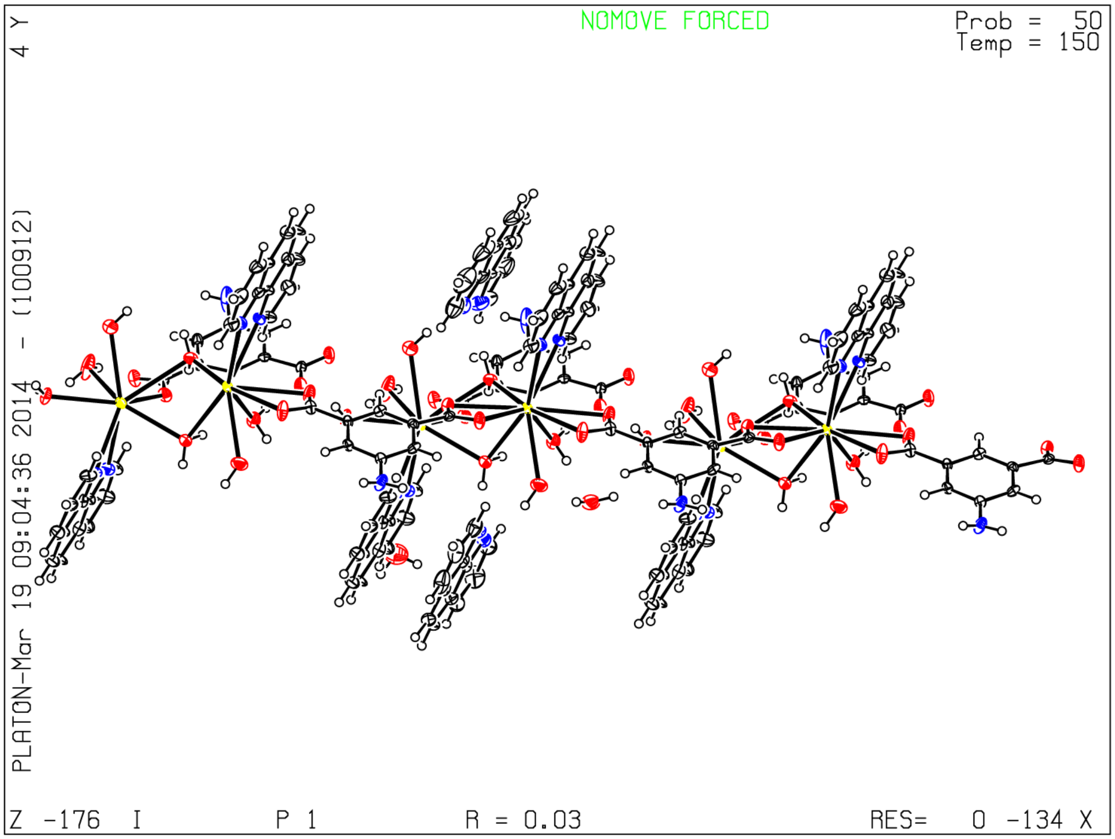

Supplement: Supplementary file 1 [file molecules-25-01353-s001.zip › 5_checkCIF.pdf]
