# Supplementary material for: From Discrete Complexes to Metal–Organic Layered Materials: Remarkable Hydrogen Bonding Frameworks
Source: Molecules. 2020 Mar 16;25(6):1353. doi: 10.3390/molecules25061353 (PMC7146137; doi:10.3390/molecules25061353)
Supplement: Supplementary file 1 [file molecules-25-01353-s001.zip › molecules-721425-supplementary material.pdf]

Electronic Supporting Information

From discrete complexes to metal-organic layered materials: remarkable hydrogen bonding frameworks

Carla Queirós, Ana M. G. Silva,

Baltazar de Castro and Luís Cunha Silva *

LAQV / REQUIMTE & Department of Chemistry and Biochemistry,

Faculty of Sciences, University of Porto, 4169-007 Porto, Portugal

* Correspondence: l.cunha.silva@fc.up.pt; Tel.: +351 220402576

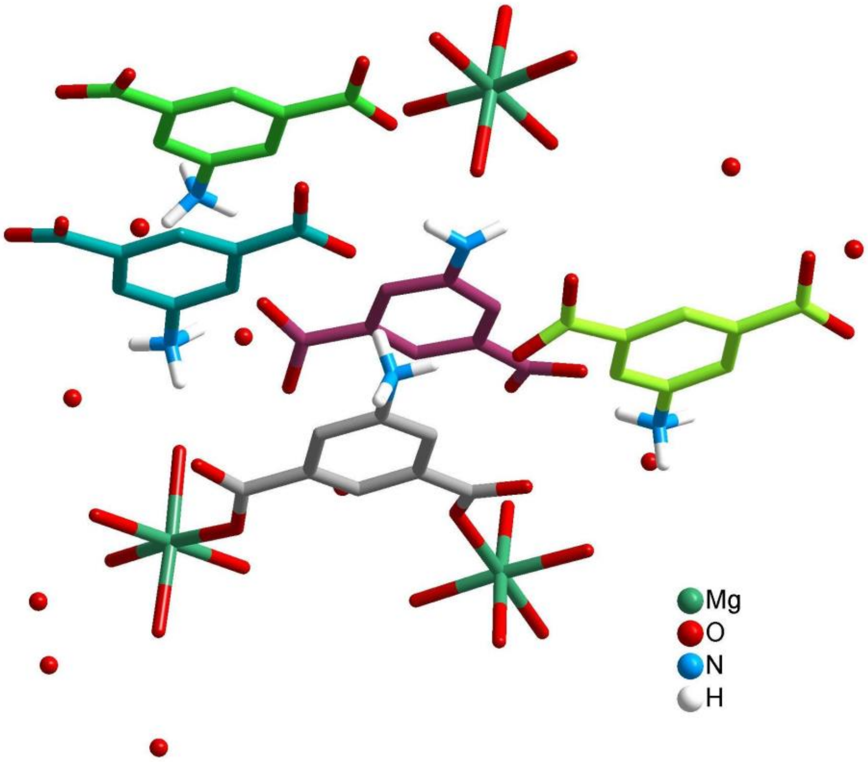

Figure S1. The asymmetric unit of the crystal structure of compound **1**, $[Mg(H_2O)_6]\cdot[Mg_2(Haip)(H_2O)_{10}]\cdot3(Haip)\cdot(aip)\cdot10(H_2O)$, with the carbon atoms of the crystallographic independent $Haip^-$ and aip^{2-} ligands drawn with different colours. For clarity reasons the *H*-atoms of the water molecules and those connected to *C*-atoms of the ligands were omitted.

Table S1. Selected distances and angles of the Mg^{2+} coordination centres of the compound **1**, $[Mg(H_2O)_6]\cdot[Mg_2(Haip)(H_2O)_{10}]\cdot3(Haip)\cdot(aip)\cdot10(H_2O)$.

**Distance /Å**		**Angles /°**		**Angles /°**		**Angles /°**	
Mg1–O1	2.036(4)	O1–Mg1–O4W	90.86(19)	O10W–Mg2–O6W	92.9(2)	O14W–Mg3–OW13	174.9(2)
Mg1–O4W	2.044(5)	O1–Mg1–O2W	97.25(19)	O10W–Mg2–O9W	89.7(2)	O14W–Mg3–OW12	88.6(2)
Mg1–O2W	2.050(5)	O1–Mg1–O1W	90.61(19)	O10W–Mg2–O3	90.79(19)	O14W–Mg3–OW16	95.2(2)
Mg1–O1W	2.057(5)	O1–Mg1–O3W	174.6(2)	O10W–Mg2–O7W	176.5(2)	O14W–Mg3–OW11	89.5(2)
Mg1–O3W	2.073(4)	O1–Mg1–O5W	86.75(19)	O10W–Mg2–O8W	87.86(19)	O14W–Mg3–OW15	87.27(19)
Mg1–O5W	2.086(5)	O4W–Mg1–O2W	89.64(19)	O6W–Mg2–O9W	176.9(2)	OW13–Mg3–OW12	95.5(2)
		O4W–Mg1–O1W	177.2(2)	O6W–Mg2–O3	87.69(19)	OW13–Mg3–OW16	88.2(2)
Mg2–O10W	2.045(5)	O4W–Mg1–O3W	87.46(19)	O6W–Mg2–O7W	90.3(2)	OW13–Mg3–OW11	87.4(2)
Mg2–O6W	2.054(5)	O4W–Mg1–O5W	91.7(2)	O6W–Mg2–O8W	90.4(2)	OW13–Mg3–OW15	88.9(2)
Mg2–O9W	2.056(5)	O2W–Mg1–O1W	87.8(2)	O9W–Mg2–O3	94.05(19)	OW12–Mg3–OW16	86.0(2)
Mg2–O3	2.059(4)	O2W–Mg1–O3W	87.83(19)	O9W–Mg2–O7W	87.1(2)	OW12–Mg3–OW11	90.6(2)
Mg2–O7W	2.063(5)	O2W–Mg1–O5W	175.8(2)	O9W–Mg2–O8W	87.90(19)	OW12–Mg3–OW15	174.6(2)
Mg2–O8W	2.070(5)	O1W–Mg1–O3W	91.3(2)	O3–Mg2–O7W	90.8(2)	OW16–Mg3–OW11	174.1(2)
		O1W–Mg1–O5W	90.7(2)	O3–Mg2–O8W	177.6(2)	OW16–Mg3–OW15	91.0(2)
Mg3–O14W	2.029(5)	O3W–Mg1–O5W	88.22(19)	O7W–Mg2–O8W	90.6(2)	OW11–Mg3–OW15	92.7(2)
Mg3–O13W	2.043(5)						
Mg3–O12W	2.057(5)						
Mg3–O16W	2.061(5)						
Mg3–O11W	2.094(5)						
Mg3–O15W	2.111(5)						

Table S2. Geometric information (distances in Å and angles in degrees) for the D–H···A hydrogen bond interactions of the $[Mg(H_2O)_6]\cdot[Mg_2(Haip)(H_2O)_{10}]\cdot(Haip)\cdot3(aip)\cdot10(H_2O)$ (**1**) crystal structure.[a]

**D–H···A**	***d* (H···A)**	***d* (D···A)**	**∠ (DHA)**
N1–H1A···O2^{i}	2.30(6)	2.923(7)	129(6)
N1–H1A···O19^{ii}	2.45(6)	3.090(7)	131(6)
N1–H2A···O9	1.84(3)	2.696(8)	168(7)
N1–H3A···O15	1.89(3)	2.765(8)	172(7)
N2–H4A···O17	2.47(6)	3.092(8)	128(6)
N2–H4A···O15	2.57(6)	3.217(8)	131(6)
N2–H5A···O8^{iii}	2.20(4)	3.004(8)	155(7)
N3–H6A···O8	2.05(5)	2.786(7)	141(6)
N3–H6A···O4	2.64(6)	3.162(7)	119(5)
N3–H7A···O12^{iv}	1.93(3)	2.802(7)	174(6)
N3–H8A···O17^{iii}	1.82(3)	2.695(7)	174(6)
N4–H9A···O12^{iii}	2.24(5)	2.947(7)	140(6)
N4–H9A···O6	2.32(6)	2.836(7)	119(5)
N4–H10A···O4^{v}	1.85(3)	2.719(7)	170(6)
N4–H11A···O13^{vi}	1.87(3)	2.710(7)	164(6)
N5–H12A···O6	1.79(3)	2.668(7)	172(6)
N5–H13A···O2	1.87(3)	2.742(7)	179(7)
N5–H14A···O13^{vi}	2.27(5)	2.846(7)	125(5)
N5–H14A···O19^{vi}	2.35(5)	2.991(7)	131(5)
O1W–H1W···O20W	1.85(2)	2.731(6)	169(6)
O1W–H2W···O5	1.89(2)	2.774(6)	171(6)
O2W–H3W···O19^{vi}	2.01(4)	2.826(6)	153(6)
O2W–H4W···O19W	1.80(2)	2.682(6)	175(7)
O3W–H5W···O24W^{vii}	1.85(3)	2.713(6)	165(6)
O3W–H6W···O17W	1.82(3)	2.692(6)	165(6)
O4W–H7W···O18W	1.84(3)	2.712(6)	157(6)
O4W–H8W···O16^{i}	1.83(3)	2.718(6)	161(5)
O5W–H9W···O22W	1.92(3)	2.795(6)	162(5)
O5W–H10W···O20W^{viii}	1.95(2)	2.837(6)	179(7)
O6W–H11W···O24W^{i}	1.97(3)	2.813(6)	157(6)
O6W–H12W···O21W^{viii}	1.95(3)	2.834(7)	172(7)
O7W–H13W···O14^{i}	1.89(3)	2.774(6)	161(7)
O7W–H14W···O21W^{ii}	1.83(3)	2.717(6)	162(7)
O8W–H15W···O22W^{viii}	1.80(3)	2.704(6)	173(5)
O8W–H16W···O25W^{ix}	1.80(3)	2.687(6)	163(6)
O9W–H17W···O4	2.03(4)	2.863(6)	155(6)
O9W–H18W···O23W	1.78(3)	2.669(6)	172(6)
O10W–H19W···O3	2.18(5)	2.922(6)	141(6)
O10W–H19W···O6W	2.38(5)	2.971(7)	124(5)
O10W–H20W···O7	1.97(5)	2.701(6)	134(6)
O11W–H21W···O16	1.92(3)	2.808(6)	171(6)
O11W–H22W···O18	2.07(4)	2.861(6)	147(5)
O12W–H23W···O18W^{i}	1.99(5)	2.793(6)	146(6)
O12W–H24W···O10^{x}	1.86(3)	2.750(6)	169(7)
O13W–H25W···O23W^{iii}	1.85(3)	2.727(6)	159(5)
O13W–H26W···O25W^{x}	1.93(3)	2.816(6)	167(7)
O14W–H27W···O17W^{i}	1.92(3)	2.813(6)	174(6)
O14W–H28W···O19W^{i}	1.86(2)	2.738(6)	165(5)
O15W–H30W···O10	2.16(4)	2.920(6)	139(5)
O16W–H31W···O18^{xi}	1.90(4)	2.734(6)	153(6)
O16W–H32W···O12W	2.10(6)	2.809(7)	135(7)
O17W–H33W···O16^{vii}	1.88(3)	2.763(6)	170(6)
O17W–H34W···O20^{vii}	1.83(3)	2.706(6)	164(6)
O18W–H35W···O3W^{viii}	2.03(3)	2.889(6)	164(6)
O18W–H35W···O2W^{viii}	2.55(6)	3.037(6)	116(4)
O18W–H36W···O20^{vii}	1.82(3)	2.704(6)	172(6)
O19W–H37W···O15^{i}	1.94(2)	2.819(6)	178(6)
O19W–H38W···O10^{i}	1.92(3)	2.782(6)	169(6)
O19W–H38W···O9^{i}	2.47(5)	3.116(7)	131(5)
O20W–H39W···O20^{vi}	2.12(4)	2.845(6)	139(5)
O20W–H39W···O18W^{xii}	2.64(3)	3.430(6)	149(5)
O20W–H40W···O14^{vi}	1.86(2)	2.747(6)	168(6)
O21W–H41W···O5	1.82(2)	2.732(6)	175(6)
O21W–H42W···O6W^{xii}	2.37(7)	2.834(7)	112(5)
O22W–H43W···O5	1.83(2)	2.709(6)	165(5)
O22W–H44W···O3	2.16(3)	3.031(6)	162(5)
O22W–H44W···O6W	2.41(5)	2.973(7)	121(4)
O23W–H45W···O18^{iii}	1.92(2)	2.804(6)	165(5)
O23W–H45W···O17^{iii}	2.41(5)	3.080(6)	131(5)
O23W–H46W···O8	1.91(4)	2.765(7)	155(6)
O24W–H47W···O6W^{i}	2.37(7)	2.813(6)	112(5)
O24W–H48W···O5W^{i}	2.21(5)	2.891(6)	135(5)
O24W–H48W···O1^{i}	2.47(3)	3.250(6)	150(5)
O25W–H49W···O13W^{xi}	2.30(5)	2.816(6)	116(4)
O25W–H50W···O7^{iii}	1.93(4)	2.759(6)	150(5)
O26W–H51W···O11	1.91(4)	2.707(6)	142(5)
O26W–H51W···O9W^{iv}	2.45(6)	2.963(7)	115(4)
O26W–H52W···O8W^{iv}	2.04(4)	2.898(6)	155(6)

[a] Symmetry transformations used to generate equivalent atoms: (i) *−x, −y+1, −z+1*; (ii) *x, y, z−1*; (iii) *−x+1, −y+1, −z+1*; (iv) *−x+1, −y+1, −z*; (v) *x, y, z+1*; (vi) *−x, −y+1, −z+2*; (vii) *−x, y+1/2, −z+3/2*; (viii) *x, −y+3/2, z−1/2*; (ix) *−x+1, y+1/2, −z+1/2*; (x) *x, −y+1/2, z+1/2*; (xi) *x, −y+1/2, z−1/2*; (xii) *x, −y+3/2, z+1/2.*

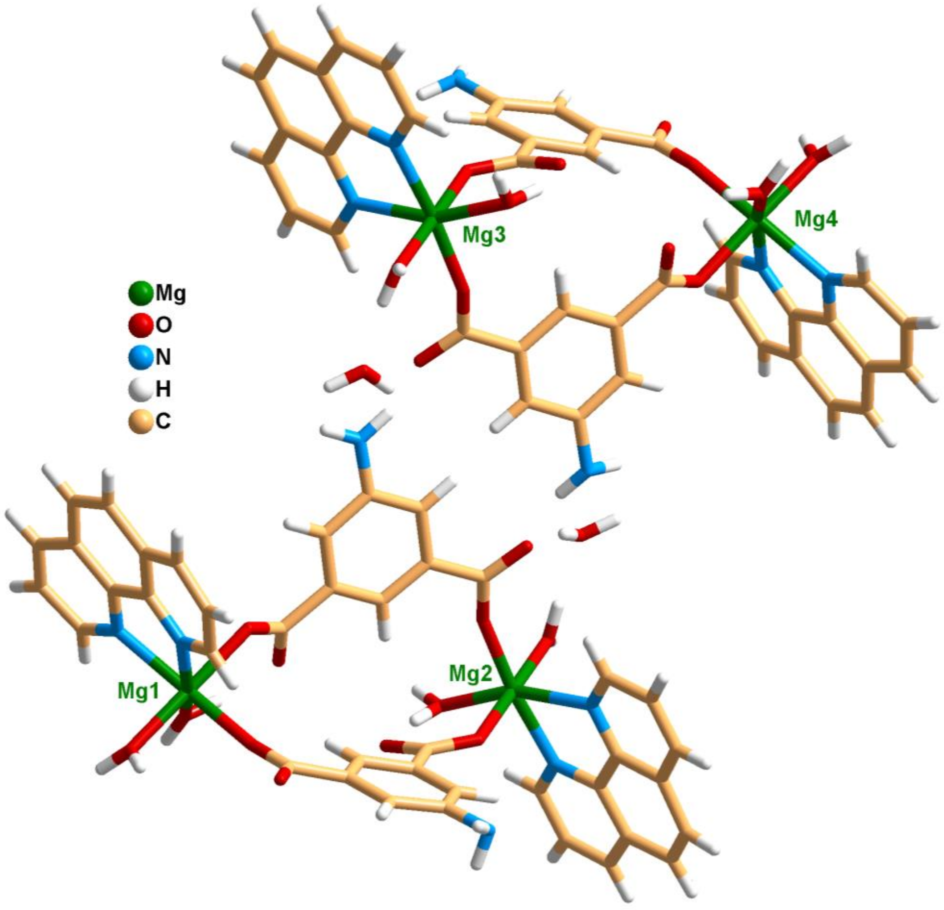

Figure S2. The asymmetric unit of the crystal structure of compound **2**, $[Mg_2(aip)_2(phen)_2(H_2O)_4]_2 \cdot 2(H_2O)$.

Table S3. Selected distances and angles of the Mg^{2+} coordination centres of the compound **2**, $[Mg_2(aip)_2(phen)_2(H_2O)_4]_2 \cdot 2(H_2O)$.

Distance /Å		Angles /°					
Mg1–O5	2.022(5)	O1–Mg1–N1	84.4(2)	O5–Mg1–O1W	89.6(2)	O1W–Mg1–O2W	93.9(2)
Mg1–O1W	2.041(5)	O1–Mg1–N2	91.2(2)	O5–Mg1–O2W	94.4(2)	O2W–Mg1–N1	164.6(2)
Mg1–O2W	2.065(5)	O5–Mg1–N1	99.3(2)	O1W–Mg1–N1	93.1(2)	O2W–Mg1–N2	91.2(2)
Mg1–O1	2.101(5)	O5–Mg1–N2	174.3(2)	O1W–Mg1–N2	89.1(2)	O2W–Mg1–O1	88.72(19)
Mg1–N2	2.193(6)	O5–Mg1–O1	89.84(19)	O1W–Mg1–O1	177.3(2)	N2–Mg1–N1	75.3(2)
Mg1–N1	2.230(6)						
Mg2–O4	2.002(5)	O4–Mg2–N5	100.5(2)	O7–Mg2–N5	80.8(2)	O4W–Mg2–N5	97.5(2)
Mg2–O4W	2.035(5)	O4–Mg2–N6	173.8(2)	O7–Mg2–N6	93.2(2)	O4W–Mg2–N6	88.2(2)
Mg2–O3W	2.055(5)	O4–Mg2–O7	90.23(19)	O3W–Mg2–N5	160.7(2)	O4W–Mg2–O7	177.4(2)
Mg2–O7	2.085(5)	O4–Mg2–O3W	94.9(2)	O3W–Mg2–N6	90.4(2)	O4W–Mg2–O3W	94.4(2)
Mg2–N6	2.205(6)	O4–Mg2–O4W	88.2(2)	O3W–Mg2–O7	87.73(19)	N6–Mg2–N5	74.9(2)
Mg2–N5	2.228(6)						
Mg3–O5W	2.041(6)	O9–Mg3–N7	101.3(2)	O13–Mg3–N8	93.2(2)	O5W–Mg3–O6W	95.4(2)
Mg3–O6W	2.075(6)	O9–Mg3–N8	175.2(3)	O15–Mg4–O8W	89.9(2)	O6W–Mg3–N7	160.4(2)
Mg3–O13	2.084(5)	O9–Mg3–O13	89.3(2)	O5W–Mg3–N7	96.9(2)	O6W–Mg3–N8	89.8(2)
Mg3–N8	2.200(6)	O9–Mg3–O5W	88.0(2)	O5W–Mg3–N8	89.2(2)	O6W–Mg3–O13	88.4(2)
Mg3–N7	2.225(6)	O13–Mg3–N7	80.1(2)	O5W–Mg3–O13	175.5(3)	N8–Mg3–N7	75.1(2)
Mg(3)-O(9)	2.015(5)						
Mg4–O15	2.026(6)	O11–Mg4–N11	90.6(2)	O15–Mg4–O7W	94.8(2)	O8W–Mg4–N11	88.7(2)
Mg4–O8W	2.031(6)	O11–Mg4–N12	84.7(2)	O7W–Mg4–N11	92.0(3)	O8W–Mg4–N12	93.9(2)
Mg4–O7W	2.063(6)	O15–Mg4–N11	173.2(3)	O7W–Mg4–N12	164.6(3)	O8W–Mg4–O11	178.6(3)
Mg4–O11	2.102(5)	O15–Mg4–N12	99.1(2)	O7W–Mg4–O11	88.5(2)	O8W–Mg4–O7W	92.8(2)
Mg4–N11	2.202(7)	O15–Mg4–O11	90.8(2)	O8W–Mg4–N11	88.7(2)	N11–Mg4–N12	74.3(3)
Mg4–N12	2.233(7)						

Table S4. Geometric information (distances in Å and angles in degrees) for the D–H···A hydrogen bond interactions of the $[Mg_2(aip)_2(phen)_2(H_2O)_4]_2 \cdot 2(H_2O)$ (**2**) crystal structure.[a]

D–H···A	*d* (H···A)	*d* (D···A)	∠ (DHA)
N3–H33A...N4^{i}	2.26	3.057(9)	150.8
N3–H33B...O10	2.33	2.945(9)	127.1
O1W–H1W...O10W^{ii}	1.80(2)	2.687(7)	172(9)
O1W–H2W...O2^{iii}	1.82(4)	2.667(6)	158(7)
O2W–H3W...O2	1.76(3)	2.596(6)	156(7)
N4–H4A...O16^{iv}	2.09(4)	2.923(9)	158(8)
O2W–H4W...O4^{iii}	2.17(5)	2.961(6)	148(7)
O3W–H5W...O8	1.73(3)	2.611(6)	165(7)
O3W–H6W...O5^{v}	2.01(3)	2.904(6)	169(7)
O4W–H7W...O8^{v}	1.79(3)	2.675(6)	169(7)
O4W–H8W...O10W	1.83(2)	2.723(7)	176(7)
N9–H5A...O6^{vi}	2.05(2)	2.929(8)	175(9)
N10–H7A...O3	2.14(4)	2.961(8)	155(7)
N10–H8A...N9^{vii}	2.41(7)	3.074(10)	131(8)
O5W–H9W...O9W	1.81(3)	2.700(7)	172(10)
O5W–H10W...O12^{viii}	1.80(4)	2.680(7)	162(8)
O6W–H11W...O14	1.81(5)	2.615(7)	148(9)
O7W–H13W...O15^{ix}	2.21(6)	2.922(7)	135(6)
O7W–H14W...O12	1.91(5)	2.605(7)	133(6)
O8W–H15W...O14^{ix}	1.76(3)	2.651(7)	165(7)
O8W–H16W...O9W^{x}	1.81(2)	2.713(8)	173(9)
O10W–H19W...O3	1.88(2)	2.756(7)	168(7)
O10W–H20W...O6^{x}	1.90(3)	2.754(7)	160(7)
O9W–H17W...O10	2.11(7)	2.770(8)	130(7)
O9W–H18W...O16^{ii}	1.89(3)	2.733(8)	160(7)

[a] Symmetry transformations used to generate equivalent atoms: (i) *x-1,y,z*; (ii) *x,y,z-1*; (iii) *x,-y+1/2,z-1/2*; (iv) *x+1,y,z-1*; (v) *x,-y+1/2,z+1/2*; (vi) *x-1,y,z+1*; (vi) *x+1,y,z*; (viii) *-x+1,-y,-z+2*; (ix) *-x+1,-y,-z+3*; (x) *x,y,z+1*.

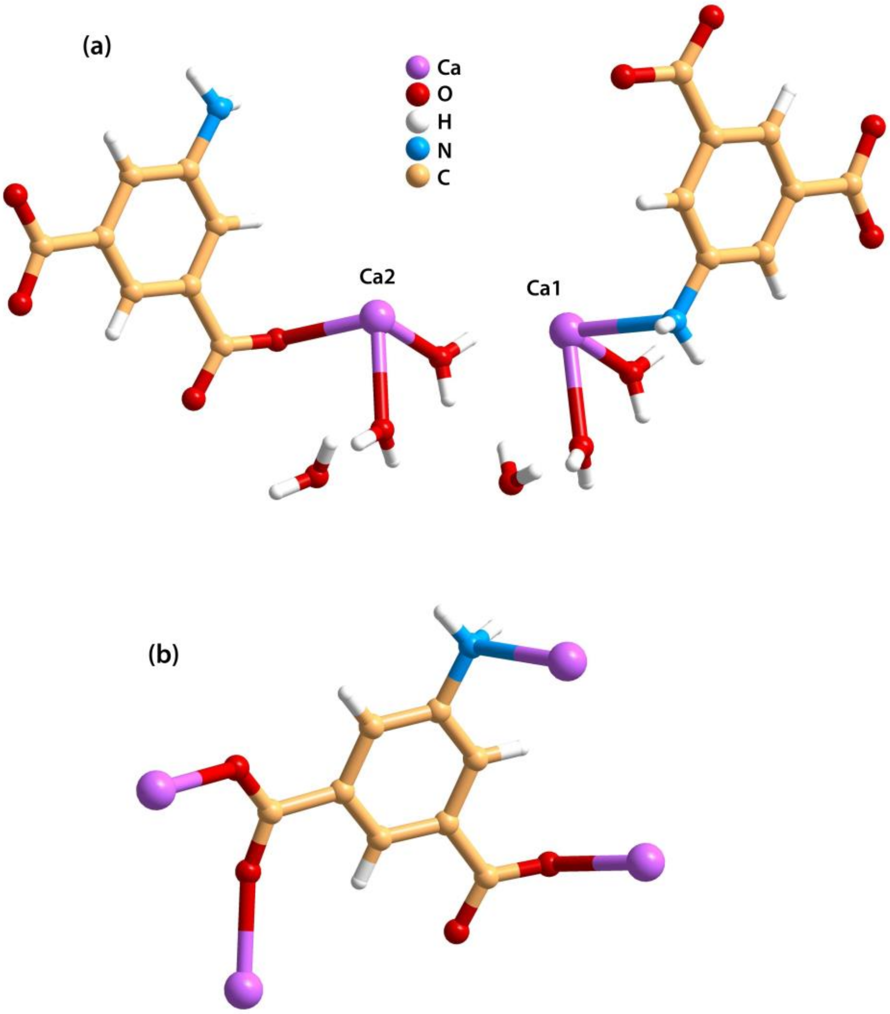

Figure S3. (**a**) The asymmetric unit of the crystal structure of compound **3**, $[Ca(aip)(H_2O)_2]\cdot(H_2O)$, represented in the ball-and-stick model. (**b**) Coordination modes of the ligand aip^{2-}.

Table S5. Selected distances and angles of the Ca^{2+} coordination centres of the compound **3**, $[Ca(aip)(H_2O)_2]\cdot(H_2O)$.[a]

Distance /Å		Angles /°		Angles /°	
Ca1–O3^{i}	2.2784(12)	O3^{i} –Ca1–O4^{ii}	103.72(5)	O7^{iv}–Ca2–O8^{v}	101.43(5)
Ca1–O4^{ii}	2.3362(13)	O3^{i} –Ca1–O1^{iii}	97.90(4)	O7^{iv}–Ca2–O6	93.46(4)
Ca1–O1^{iii}	2.3719(13)	O3^{i} –Ca1–O1W	172.27(5)	O7^{iv}–Ca2–O3W	175.79(5)
Ca1–O1W	2.3788(13)	O3^{i} –Ca1–O2W	97.47(5)	O7^{iv}–Ca2–O4W	96.69(5)
Ca1–O2W	2.3980(13)	O3^{i} –Ca1–N1	93.52(5)	O7^{iv}–Ca2–N2^{vi}	95.33(5)
Ca1–N1	2.5446(17)	O4^{ii} –Ca1–O1^{iii}	87.25(5)	O8^{v}–Ca2–O6	89.21(5)
		O4^{ii} –Ca1–O1W	82.07(5)	O8^{v}–Ca2–O3W	82.74(5)
Ca2–O7^{iv}	2.2582(12)	O4^{ii}–Ca1–O2W	158.27(5)	O8^{v}–Ca2–O4W	160.92(5)
Ca2–O8^{v}	2.3055(13)	O4^{ii} –Ca1–N1	83.05(5)	O8^{v}–Ca2–N2^{vi}	169.59(5)
Ca2–O6	2.3684(13)	O1^{iii} –Ca1–O1W	87.40(5)	O6–Ca2–O3W	87.07(5)
Ca2–O3W	2.3837(13)	O1^{iii}–Ca1–O2W	94.62(5)	O6–Ca2–O4W	95.64(5)
Ca2–O4W	2.3887(13)	O1^{iii} –Ca1–N1	166.49(5)	O6–Ca2–N2^{vi}	169.59(5)
Ca2–N2^{vi}	2.5672(17)	O1W–Ca1–O2W	76.40(5)	O3W–Ca2–O4W	79.10(5)
		O1W –Ca1–N1	81.98(5)	O3W–Ca2–N2^{vi}	84.57(5)
		O2W –Ca1–N1	90.99(5)	O4W–Ca2–N2^{vi}	88.86(5)

[a] Symmetry transformations used to generate equivalent atoms: (i) *−x+1, −y+2, −z+1;* (ii) *x−1,y,z;* (iii) *−x, −y+2, −z+1;* (iv) *x+1,y,z;* (v) *−x−1, −y+2, −z*; (vi) *−x, −y+2, −z*

Table S6. Geometric information (distances in Å and angles in degrees) for the D–H···O hydrogen bond interactions of the [Ca(aip)(H_2O)$_2$]·(H_2O) crystal structure (compound **3**).[a]

D–H···O	*d* (H···O)	*d* (D···O)	∠(DHO)
O1W–H1W···O5W	2.066(17)	2.847(2)	153(2)
O1W–H2W···O1^{vii}	1.853(16)	2.7623(18)	171(2)
N1–H1A···O6W^{viii}	2.137(15)	3.014(2)	176.3(19)
N1–H2A···O6^{ix}	2.352(17)	3.169(2)	157.1(18)
O2W–H3W···O2^{i}	2.002(16)	2.8653(19)	166(2)
O2W–H4W···O2^{vii}	1.904(16)	2.7742(18)	166(2)
O3W–H5W···O6W	1.910(16)	2.771(2)	172(2)
O3W–H6W···O6^{x}	1.962(16)	2.8226(18)	171(2)
N2–H3A···O1^{11}	2.375(16)	3.209(2)	159.6(18)
N2–H4A···O5W^{xii}	2.274(15)	3.155(2)	170.1(18)
O4W–H7W···O5^{iv}	2.000(16)	2.8825(19)	172(2)
O4W–H8W···O5^{x}	1.876(17)	2.7308(17)	161(2)
O5W–H9W···O1W	2.45(3)	2.847(2)	111(2)
O5W–H9W···O4^{ii}	2.37(2)	3.097(2)	150(2)
O5W–H10W···O5^{ix}	1.892(17)	2.768(2)	173(3)
O6W–H11W···O2^{iii}	2.10(2)	2.836(2)	143(2)
O6W–H12W···O5W^{xiii}	1.858(16)	2.774(3)	175.2(19)

[a] Symmetry transformations used to generate equivalent atoms:
(i)*−x+1, −y+2, −z+1;* (ii) *x−1, y, z;* (iii) *−x, −y+2, −z+1*; (iv) *x+1, y, z*; (v) *−x−1, −y+2, −z*; (vi) *−x, −y+2, −z*; (vii) *x, y−1, z*; (viii) *−x, −y+1, −z+1*; (ix) *x, y, z+1*; (x) *−x, −y+1, −z*; (xi) *x, y, z−1*; (xii) *x, y+1, z−1*; (xiii) *−x−1, −y+1, −z+1*.

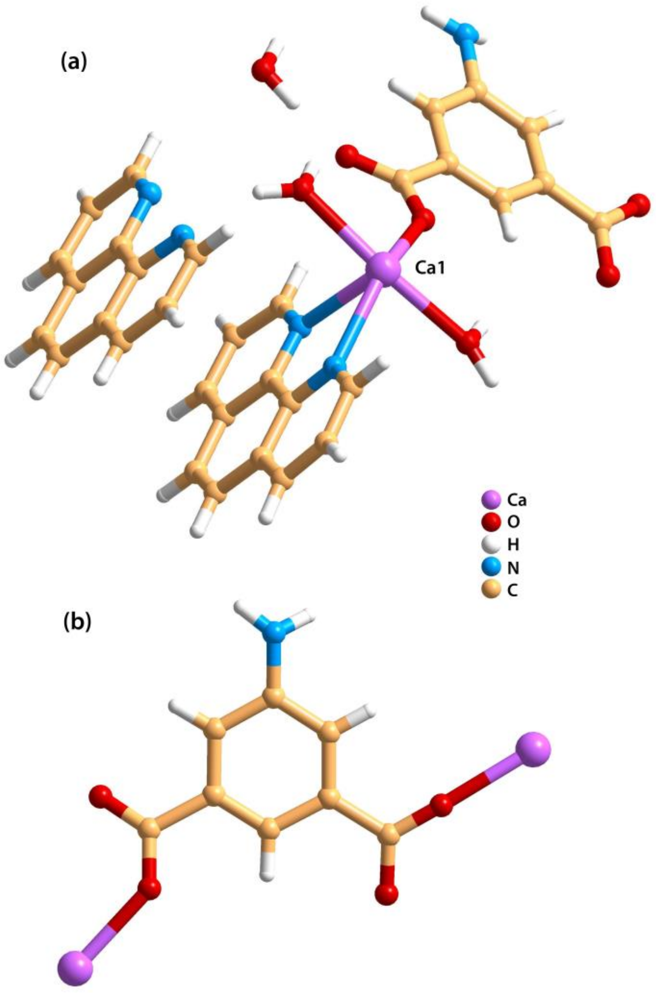

Figure S4. (**a**) The asymmetric unit of the crystal structure of the metal-organic chain **4**, [Ca(aip)(phen)(H_2O)$_2$]·(phen)·(H_2O), represented in the ball-and-stick model. (**b**) Coordination modes of the ligand aip^{2-}.

Table S7. Selected distances and angles of the Ca^{2+} coordination centre of the compound **4**, [Ca(aip)(phen)(H_2O)$_2$]·(phen)·(H_2O).

**Distance /Å**		**Angles /°**	
Ca1–O4^{i}	2.249(4)	O4^{i}–Ca1–O1	115.29(14)
Ca1–O1	2.282(4)	O4^{i}–Ca1–O1W	96.95(15)
Ca1–O1W	2.366(4)	O4^{i}–Ca1–O2W	97.87(14)
Ca1–O2W	2.388(4)	O4^{i}–Ca1–N2	152.03(15)
Ca1–N2	2.501(5)	O4^{i}–Ca1–N1	91.02(15)
Ca1–N1	2.534(5)	O1–Ca1–O1W	82.18(14)
		O1–Ca1–O2W	80.19(14)
		O1–Ca1–N2	91.14(14)
		O1–Ca1–N1	150.86(15)
		O1W–Ca1–O2W	160.59(15)
		O1W–Ca1–N2	95.53(15)
		O1W–Ca1–N1	82.38(15)
		O2W–Ca1–N2	76.83(14)
		O2W–Ca1–N1	109.77(15)
		N2–Ca1–N1	65.98(15)

Symmetry transformations used to generate equivalent atoms: (i) *−x, y+1/2, −z+2*

Table S8. Geometric information (distances in Å and angles in degrees) for the D–H···O hydrogen bond interactions of the [Ca(aip)(phen)(H_2O)$_2$]·(phen)·(H_2O) crystal structure (**4**).[a]

**D–H···O**	***d*(H···A)**	***d*(D···A)**	**∠(DHA)**
O1W–H1W···O3W^{iii}	2.04(4)	2.802(6)	141(4)
O1W–H1W···O1	2.60(5)	3.056(5)	112(4)
O2W–H3W···O2	1.81(3)	2.650(6)	156(5)
O2W–H4W···N3	1.94(3)	2.750(6)	153(4)
N5–H1A···O2W^{iv}	2.14(4)	2.887(6)	144(5)
N5–H2A···O3^{v}	2.14(3)	2.950(6)	154(5)
N5–H2A···O4^{v}	2.48(4)	3.262(6)	149(5)
O3W–H5W···O2	1.85(3)	2.714(5)	158(6)
O3W–H6W···O3^{v}	1.99(3)	2.865(5)	165(6)

[a] Symmetry transformations used to generate equivalent atoms: (i) *−x, y+1/2, −z+2;* (ii) *−x, y−1/2, −z+2;* (iii) *x−1, y, z;* (iv) *−x+1, y−1/2 , −z+2* (v) *x+1, y, z*

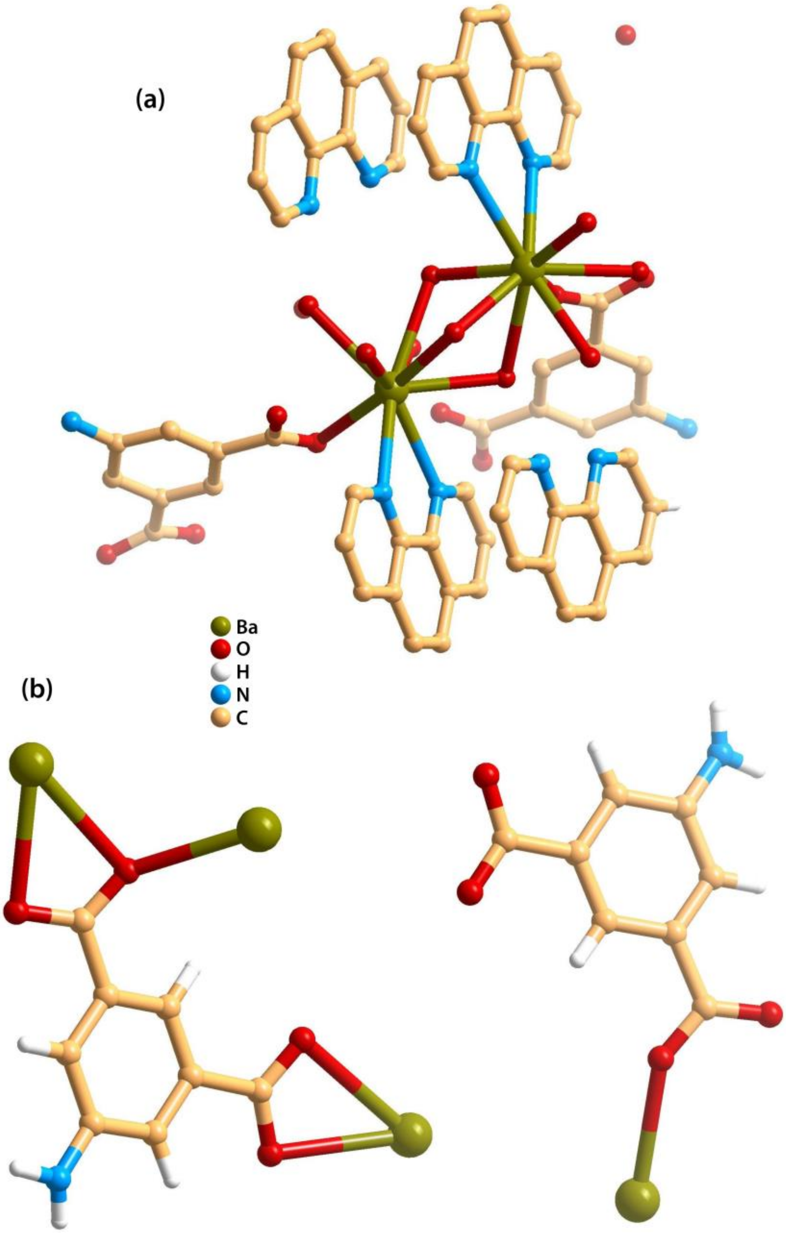

Figure S5. (**a**) The asymmetric unit of the crystal structure of compound **5**, $[Ba_2(aip)_2(phen)_2(H_2O)_7]\cdot 2(phen)\cdot 2(H_2O)$, represented in the ball-and-stick model. (**b**) Coordination modes of the two crystallographic independent aip^{2-} ligands.

Table S9. Selected distances and angles of the Ba^{2+} coordination centres of compound **5** [$Ba_2(aip)_2(phen)_2(H_2O)_7$]·2(phen)·2($H_2O$).

Distance /Å		Angles /°							
Ba(1)–O(7)^{i}	2.726(2)	O7–Ba1–O1W^{i}	136.06(10)	O2W–Ba1–O1	133.73(10)	O5W–Ba2–N6	105.56(11)	N5–Ba2–N6	55.89(13)
Ba(1)–O(1W)	2.738(3)	O7–Ba1–O4W^{i}	70.90(7)	O2W–Ba1–O3W	76.40(11)	O5W–Ba2–O6W	67.24(7)	N5–Ba2–O6W	87.94(10)
Ba(1)–O(4W)	2.748(2)	O7–Ba1–O2W^{i}	100.22(9)	O2W–Ba1–N4	72.45(12)	O5W–Ba2–O6	119.19(8)	N5–Ba2–O6	70.68(12)
Ba(1)–O(2W)	2.780(3)	O7–Ba1–O1^{i}	119.69(10)	O2W–Ba1–N3	90.54(13)	O5W–Ba2–O4W	63.35(7)	N5–Ba2–O4W	127.33(10)
Ba(1)–O(1)	2.794(4)	O7–Ba1–O3W^{i}	74.78(10)	O2W–Ba1–O5W	141.62(9)	O8–Ba2–O5^{i}	83.16(10)	O7W–Ba2–O7^{I}	98.97(10)
Ba(1)–O(3W)	2.807(4)	O7–Ba1–N4^{i}	90.13(10)	O1–Ba1–O3W	91.56(11)	O8–Ba2–N5^{i}	127.97(12)	O7W–Ba2–N6	128.65(12)
Ba(1)–N(4)	2.915(5)	O7–Ba1–N3^{i}	139.92(11)	O1–Ba1–N4	125.56(12)	O8–Ba2–O7W^{i}	77.94(12)	O7W–Ba2–O6W	73.89(10)
Ba(1)–N(3)	2.937(5)	O7–Ba1–O5W^{i}	66.83(7)	O1–Ba1–N3	74.23(13)	O8–Ba2–O7^{i}	46.11(9)	O7W–Ba2–O6	76.70(11)
Ba(1)–O(5W)	3.017(2)	O1W–Ba1–O4W	137.25(9)	O1–Ba1–O5W	54.45(9)	O8–Ba2–N6^{i}	75.70(13)	O7W–Ba2–O4W	62.93(8)
		O1W–Ba1–O2W	69.83(10)	O3W–Ba1–N4	142.21(12)	O8–Ba2–O6W^{i}	142.74(10)	O7–Ba2–N6i	93.37(11)
Ba(2)–O(5W)	2.823(2)	O1W–Ba1–O1	65.33(10)	O3W–Ba1–N3	145.15(13)	O8–Ba2–O6^{i}	127.78(10)	O7–Ba2–O6W^{i}	115.48(7)
Ba(2)–O(8)^{i}	2.846(4)	O1W–Ba1–O3W	61.29(12)	O3W–Ba1–O5W	65.43(9)	O8–Ba2–O4W^{i}	90.83(9)	O7–Ba2–O6^{i}	173.64(8)
Ba(2)–O(5)	2.851(3)	O1W–Ba1–N4	123.86(12)	N4–Ba1–N3	56.41(14)	O5–Ba2–N5	100.13(11)	O7–Ba2–O4W^{i}	65.06(7)
Ba(2)–N(5)	2.855(4)	O1W–Ba1–N3	83.92(13)	N4–Ba1–O5W	139.50(10)	O5–Ba2–O7W	58.44(9)	N6–Ba2–O6W	141.48(11)
Ba(2)–O(7W)	2.887(4)	O1W–Ba1–O5W	94.12(9)	N3–Ba1–O5W	123.22(11)	O5–Ba2–O7^{I}	129.02(8)	N6–Ba2–O6	85.92(12)
Ba(2)–O(7)^{i}	2.916(2)	O4W–Ba1–O2W	148.80(9)			O5–Ba2–N6	75.20(11)	N6–Ba2–O4W	158.01(11)
Ba(2)–N(6)	2.956(5)	O4W–Ba1–O1	72.22(10)	O5W–Ba2–O8^{i}	112.79(9)	O5–Ba2–O6W	101.82(8)	O6W–Ba2–O6	68.09(8)
Ba(2)–O(6W)	2.975(3)	O4W–Ba1–O3W	126.46(9)	O5W–Ba2–O5	163.88(8)	O5–Ba2–O6	44.72(9)	O6W–Ba2–O4W	54.69(7)
Ba(2)–O(6)	2.981(4)	O4W–Ba1–N4	77.62(10)	O5W–Ba2–N5	68.64(9)	O5–Ba2–O4W	121.03(8)	O6–Ba2–O4W	116.00(9)
Ba(2)–O(4W)	2.987(2)	O4W–Ba1–N3	79.97(11)	O5W–Ba2–O7W	125.20(8)	N5–Ba2–O7W	146.75(12)		
		O4W–Ba1–O5W	63.77(7)	O5W–Ba2–O7^{i}	67.09(7)	N5–Ba2–O7^{I}	114.06(10)		

Symmetry transformations used to generate equivalent atoms: (i) *x+1, y, z*

Table S10. Geometric information (distances in Å and angles in degrees) for the D–H···A hydrogen bond interactions of the $[Ba_2(aip)_2(phen)_2(H_2O)_7]\cdot 2(phen)\cdot 2(H_2O)$ crystal structure (compound **5**).[a]

D–H···O	*d* (H···A)	*d* (D···A)	∠(DHA)
N1–H1A···O8^{iii}	2.14(5)	2.847(5)	140(5)
N(1)–H(2A)···O(5)^{iv}	2.04(3)	2.858(5)	160(6)
N(2)–H(3A)···O(2)^{v}	2.18(3)	2.996(6)	155(4)
N(2)–H(4A)···O(4)vi	2.29(3)	3.104(6)	158(4)
O1W–H2W···O2W	2.61(5)	3.158(5)	122(4)
O1W–H1W···O2	1.88(3)	2.712(4)	157(5)
O2W–H3W···O3^{i}	2.00(3)	2.763(5)	150(4)
O2W–H3W···O1W	2.66(5)	3.158(5)	119(4)
O2W–H4W···O6^{i}	1.918(19)	2.774(4)	167(4)
O3W–H5W···N10	2.00(2)	2.845(6)	163(5)
O3W–H5W···N9	2.68(4)	3.263(7)	126(4)
O3W–H6W···O1W	2.14(4)	2.827(5)	132(5)
O4W–H7W···O6W	1.96(3)	2.739(4)	145(4)
O4W–H8W···N7	1.97(2)	2.837(5)	169(3)
O5W–H9W···N9	2.10(2)	2.967(6)	160(3)
O5W–H9W···N10	2.70(3)	3.363(6)	131(3)
O5W–H10W···O1	1.84(2)	2.666(4)	156(4)
O6W–H11W···O8W	1.936(19)	2.809(5)	170(4)
O6W–H12W···O3	1.82(2)	2.720(5)	164(4)
O7W–H13W···N8	2.01(3)	2.856(7)	154(4)
O7W–H14W···O8W	2.15(5)	2.840(7)	131(5)
O7W–H14W···O4W	2.63(6)	3.067(4)	110(5)
O8W–H15W···O7W	1.96(3)	2.840(7)	160(5)
O8W–H16W···O2W^{ii}	2.65(5)	3.160(6)	117(4)
O9W–H17W···O3W^{vii}	1.83(3)	2.719(7)	158(7)
O9W–H18W···O6^{viii}	2.16(3)	3.076(7)	168(6)

[a] Symmetry transformations used to generate equivalent atoms:
(i) *x+1, y, z;* (ii) *x−1, y, z;* (iii) *x+1, y−1, z+1;* (iv) *x, y−1, z+1;* (v) *x−1, y+1, z−1*;
(vi) *x, y+1, z−1;* (vii) *x, y−1, z;* (viii) *x+1, y−1, z.*

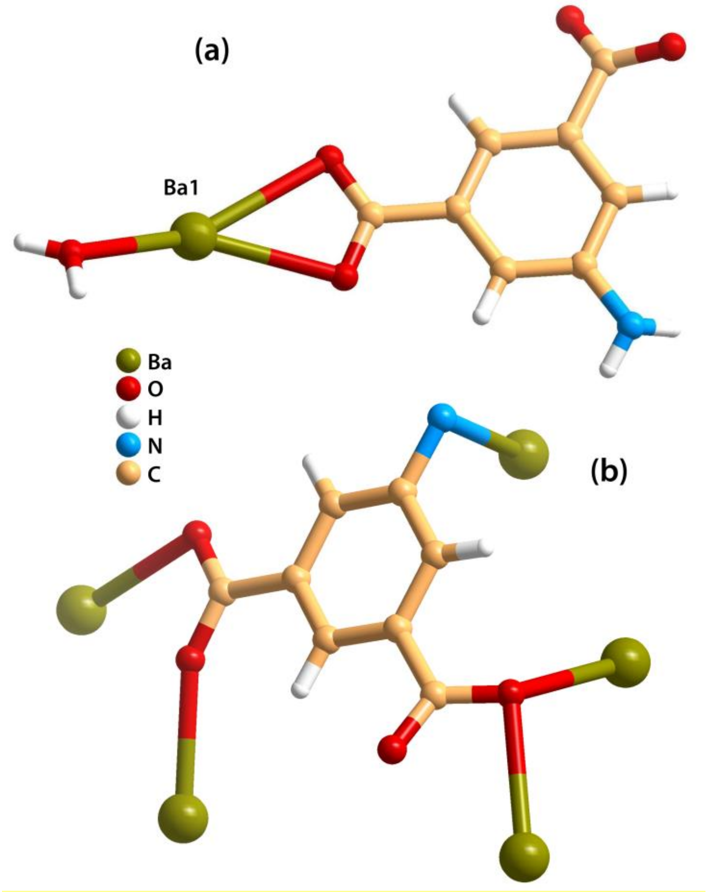

Figure S6. (**a**) The asymmetric unit of the crystal structure of compound **6**, [Ba(aip)(H_2O)], represented in the ball-and-stick model. (**b**) Coordination modes of the ligand aip^{2-} found in the crystal of **6**.

Table S11. Selected distances and angles of the Ba^{2+} coordination centre of the compound **6,** [Ba(aip)(H_2O)].

**Distance /Å**		**Angles /°**	
Ba1–O2^{i}	2.6802(14)	O2^{i}–Ba1–O1W	89.62(5)
Ba1–O1W	2.7276(15)	O2^{i}–Ba1–O1	117.49(4
Ba1–O1	2.7456(14)	O2^{i}–Ba1–O4^{ii}	105.02(5)
Ba1–O4^{ii}	2.7594(15)	O2^{i}–Ba1– O4^{iii}	167.88(4)
Ba1–O4^{iii}	2.8321(14)	O2^{i}–Ba1– O3^{iii}	126.74(4)
Ba1–O3^{iii}	2.8815(14)	O2^{i}–Ba1–N1^{iv}	98.12(5)
Ba1–N1^{iv}	2.9255(19)	O1W–Ba1–O1	143.23(4)
		O1W–Ba1–O4^{ii}	70.96(4)
		O1W–Ba1– O4^{iii}	78.55(4)
		O1W–Ba1– O3^{iii}	67.07(5)
		O1W–Ba1–N1^{iv}	126.21(5)
		O1–Ba1–O4^{ii}	77.89(4)
		O1–Ba1–O4^{iii}	74.32(4)
		O1–Ba1–O3^{iii}	107.97(4)
		O1–Ba1–N1^{iv}	76.83(5)
		O4^{ii}–Ba1–O4^{iii}	73.74(5)
		O4^{ii}–Ba1–O3^{iii}	110.48(4)
		O4^{ii}–Ba1–N1^{iv}	151.54(5)
		O4^{iii}–Ba1–O3^{iii}	45.82(4)
		O4^{iii}–Ba1–N1^{iv}	86.97(4)
		O3^{iii}–Ba1–N1^{iv}	65.96(5)

Symmetry transformations used to generate equivalent atoms: (i) *−x, −y+1, −z+1;* (ii) *−x, −y, −z+1;* (iii) *x, y+1, z−1*; (iv) *x, y+1, z*.

Table S12. Geometric information (distances in Å and angles in degrees) for the D–H···O hydrogen bond interactions of the [Ba(aip)(H_2O)] crystal structure (**6**).[a]

**D–H···O**	***d*(H···A)**	***d*(D···A)**	**∠(DHA)**
O1W–H1W···O3^{i}	1.898(16)	2.762(2)	173(3)
O1W–H2W···O1^{ii}	1.884(16)	2.762(2)	174(2)
N1–H1A···O1^{iii}	2.35(2)	3.058(2)	140(2)
N1–H2A···O4^{iv}	2.338(18)	3.145(2)	159(2)

[a] Symmetry transformations used to generate equivalent atoms:
(i) *−x, −y+1, −z+1*; (ii) *−x, −y+1, −z;* (iii) *−x+1, −y, −z;* (iv) *−x+1, −y−1, −z+1*
